# Ming-Mu-Di-Huang-Pill Activates SQSTM1 via AMPK-Mediated Autophagic KEAP1 Degradation and Protects RPE Cells from Oxidative Damage

**DOI:** 10.1155/2022/5851315

**Published:** 2022-03-25

**Authors:** Xi Chen, Yujie Zhu, Xiaoqing Shi, Jing Zuo, Tianming Hu, Hao Wu, Ying Xia, Wei Shi, Wei Wei

**Affiliations:** ^1^First College of Clinical Medicine, Nanjing University of Chinese Medicine, Nanjing, Jiangsu 210029, China; ^2^Department of Ophthalmology, The Affiliated Hospital of Nanjing University of Chinese Medicine, Nanjing, Jiangsu 210029, China; ^3^Key Laboratory for Metabolic Diseases in Chinese Medicine, First College of Clinical Medicine, Nanjing University of Chinese Medicine, Nanjing, Jiangsu 210029, China

## Abstract

Oxidative stress and diminished autophagy in the retinal pigment epithelium (RPE) play crucial roles in the pathogenesis of age-related macular degeneration (AMD). Enhancing autophagy has recently been identified as an important strategy to protect RPE cells from oxidative damage. Ming-Mu-Di-Huang-Pill (MMDH pill) is a traditional herbal medicine used to treat AMD, and its molecular mechanism is not well understood. The aim of the present study was to investigate whether the MMDH pill relieved acute oxidative damage by activating autophagy in an in vitro and in vivo model of sodium iodate (NaIO_3_). The results showed that NaIO_3_ induced cell death and inhibited proliferation. The MMDH pill increased cell viability, restored the activities of antioxidant enzymes, and reduced reactive oxygen species (ROS) fluorescence intensity. The MMDH pill mediated Kelch-like ECH-associated protein 1 (Keap1) degradation and decreased oxidative damage, which was blocked in autophagy inhibitor (chloroquine) or sequestosome-1 (SQSTM1) siRNA-treated RPE cells. Furthermore, we indicated that the MMDH pill could promote adenosine monophosphate-activated protein kinase (AMPK) phosphorylation and autophagy adaptor-SQSTM1 expression, which could stimulate autophagic degradation of Keap1. In addition, the MMDH pill increased nuclear factor (erythroid-derived 2)-like 2 (Nrf2) nuclear translocation in a SQSTM1-dependent manner and induced the expression of the downstream antioxidant factors heme oxygenase-1 (HO-1) and nicotinamide adenine dinucleotide phosphate quinone dehydrogenase 1 (NQO1). In conclusion, MMDH pill plays a protective role in relieving NaIO_3_-induced oxidative stress by activating the AMPK/SQSTM1/Keap1 pathway. The MMDH pill may be useful to treat AMD by maintaining redox homeostasis and autophagy.

## 1. Introduction

Age-related macular degeneration (AMD) is a leading irreversible and severe visual loss disease in older persons in industrialized nations. It has been suggested that the pathogenesis of AMD involves oxidative and endoplasmic reticulum (ER) stress, impaired autophagy, mitochondrial dysfunction, and inflammation [1–6]. The retinal pigment epithelium (RPE) is the primary pathological site in AMD. Oxidative stress to the RPE has been acknowledged to be the key risk factor for AMD pathogenesis and progression [7]. It has been reported that the RPE of donors with AMD includes a high content of SQSTM1, contributing to dysregulated autophagy [8]. Generally, oxidative stress and autophagy can be therapeutic targets for AMD treatment [9].

The Kelch-like ECH-associated protein 1- (Keap1-) nuclear factor E2-related factor 2 (Nrf2) pathway is a key regulator of cellular defense against oxidative stress. In the presence of oxidative stress, Nrf2 dissociates from KEAP1, translocates into the nucleus, and subsequently induces antioxidant genes [10]. Sequestosome-1 (SQSTM1/p62) serves as a selective autophagy receptor that is involved in the targeting of aggregation-prone proteins and damaged organelles into autophagosomes for degradation via the lysosome [11]. Multiple studies have shown that aggregation of SQSTM1 enhances its interaction with Keap1, binds and recruits Keap1 to autophagosomes, and subsequently activates translocation of Nrf2 to the nucleus [12–14]. As an energy sensor, adenosine monophosphate-activated protein kinase (AMPK) plays an important role in activating autophagy by inhibiting mammalian target of rapamycin (mTOR) [15]. Although AMPK is a key modulator of autophagy, it is less clear whether AMPK is involved in SQSTM1-dependent autophagy to initiate clearance of Keap1 in RPE cells.

Traditional Chinese medicine (TCM) theories have shown that the pathogenesis of AMD lies in liver-kidney Yin deficiency. The MMDH pill is a representative and classic TCM prescription for treating AMD and was first recorded in the book “Shenshi Yaohan” during the Ming dynasty. According to TCM theory, the MMDH pill contains 11 herbs, possessing the functions of nourishing the kidney, calming the liver, and improving eyesight. Our previous study indicated that Fructus lycii, Rehmanniae Radix Praeparata, and Paeonia lactiflora (the main three herbs of the MMDH pill) reduce H_2_O_2_-induced oxidative stress and apoptosis in RPE cells [16].

However, the mechanisms of treating AMD in MMDH pills have not been comprehensively investigated. A previous study demonstrated that sodium iodate (NaIO_3_) can induce RPE cell damage, imitating in vitro and in vivo models of AMD [17]. In this study, we investigated the pharmacological actions of the MMDH pill and attempted to define the role of AMPK in the regulation of the SQSMT1-Keap1-Nrf2 feedback loop in oxidative stress. This hypothesis was tested both in AMD mice and in RPE cells exposed to NaIO_3_.

## 2. Methods

### 2.1. Preparation of the MMDH Pill and UPLC-QTOF-MS Analysis

According to the experimental design, *Rehmanniae Radix Praeparata* 24 g, *Cornus officinalis* 12 g, *cortex moutan* 9 g, *Dioscoreae Rhizoma* 12 g, *Poria cocos* 9 g, *Alismatis rhizome* 9 g, *Lycium barbarum* 12 g, *Chrysanthemum morifolium* 9 g, *Angelica sinensis* 12 g, *Radix paeoniae alba* 12 g, and *Tribulus terrestris L* 9 g were purchased from the affiliated hospital of Nanjing University of Chinese Medicine. The herbs were mixed, and the mixture was decocted with distilled water and then filtered. Ultrahigh-performance liquid chromatography tandem mass spectrometry (UHPLC-MS/MS) analysis was performed on an UHPLC system (Vanquish, Thermo Fisher Scientific) with a Waters UPLC BEH C18 column (1.7 *μ*m 2.1∗100 mm). The mobile phase consisted of 0.1% formic acid in water (A) and 0.1% formic acid in acetonitrile (B). A gradient elution program was applied (0-11 min, 85-25% A; 11-12 min, 25-2% A; 12-14 min, 2-2% A; 14-14.1 min, 2-85% A; 14.1-16 min, 85%-85% A). The flow rate was 500 *μ*L/min, and the injected sample volume was 5 *μ*L. A characteristic acquire mode IDA was employed to gain the MS/MS information of ions when acquiring MS information. During each acquisition cycle, the mass range was from 100 to 1500, and the top four of every cycle were screened and the corresponding MS/MS data were further acquired. Sheath gas flow rate: 35 Arb, Aux gas flow rate: 15 Arb, ion transfer tube temp: 350°C, vaporizer temp: 350°C, full ms resolution: 60000, MS/MS resolution: 15000, collision energy: 16/38/42 in NCE mode, spray voltage: 5.5 kV (positive) and -4 kV (negative). The MMDH pill was used at two doses in an in vivo experiment: low dose (0.22 g/mL) and high dose (0.44 g/mL). Finally, the filtrate was prepared by freeze-drying for further in vitro experiments.

### 2.2. Animal Experiments

Sixty male C57BL/6 mice (10-12 weeks old, 25–30 g) were raised in the experimental animal center of Nanjing University of Chinese Medicine with free access to food and water. All animal procedures adhered to the ethics committee of Nanjing University of Chinese Medicine. The sodium iodate (NaIO_3_; Sigma-Aldrich Corp., St. Louis, MO, USA) was dissolved in sterile saline. The mice were randomly allocated into four groups: normal, NaIO_3_, low-dose MMDH pill+NaIO_3_, and high-dose MMDH pill+NaIO_3_ groups. The mice in the sham and NaIO_3_ groups were given 0.2 mL of saline (once a day) by gavage for 30 days. The mice in the MMDH pill+NaIO_3_ pill groups were pretreated 0.2 mL MMDH pill solution (0.22 or 0.44 g/mL, once a day) by gavage for 30 days and then were injected with 30 mg/kg NaIO_3_ through a tail vein. All mice were sacrificed at 7 days after injection.

### 2.3. Cell Culture and Treatments

The RPE cell line (ARPE-19) was cultured in Dulbecco's modified Eagle's medium (DMEM)/F-12 (Thermo Fisher Scientific) supplemented with 10% fetal bovine serum (FBS; Thermo Fisher Scientific), 100 U/mL penicillin, and 100 *μ*g/mL streptomycin in an atmosphere of 5% CO_2_ at 37°C. RPE cells were pretreated with various doses of MMDH pill freeze-dried powder (0.005-0.05 mg/mL) for 24 h and then treated with NaIO_3_ (10 mM) for 24 h.

### 2.4. Cell Viability Assay

Cell viability assays were performed by using Cell Counting Kit-8 (CCK8; Biosharp, Hefei, China) according to the manufacturer's instructions. RPE cells were seeded in a 96-well plate at a density of 4 × 10^3^ cells/well and treated with MMDH pills and NaIO_3_ for the indicated times. Following drug treatment, CCK8 (10 *μ*L) was added to each well and incubated at 37°C for another 2 h. The absorbance at 450 nm was measured on a microplate reader. All experiments were conducted independently in triplicate.

### 2.5. Mitochondrial Membrane Potential (ΔΨ*m*) Assay

The collapse of mitochondrial membrane potential (MMP) function is an indicator event in the early stage of apoptosis. We prepared JC-1 fluorescent probes (Beyotime, China) to measure the changes in MMP according to the manufacturer's instructions. RPE cells were treated with the designated conditions and then incubated with 1 mL JC-1 solution for 30 min at 37°C in the dark. The ratio of red fluorescence to green fluorescence was monitored by fluorescence microscopy.

### 2.6. Detection of Intracellular ROS Accumulation

Intracellular reactive oxygen species (ROS) levels were assessed via the probe 2′,7′-dichlorofluorescein-diacetate (DCFH-DA, Sigma-Aldrich, MO, USA). Various groups of RPE cells were incubated with 10 *μ*M DCFH-DA at 37°C for 30 min. The level of ROS produced in RPE cells was measured by fluorescence microscopy at an emission wavelength of 530 nm and excitation wavelength of 485 nm.

### 2.7. Small Interfering RNA Transfection

siRNA sequences targeting human SQSTM1 and a negative control (si-NC) were constructed by GenePharma (Shanghai, China). RPE cells in each well were transfected with si-NC and si-SQSTM1 with the help of Lipofectamine 2000 (Invitrogen, CA) according to the manufacturer's instructions. After 6 h of incubation, the medium was replaced with DMEM containing 10% FBS per well, followed by further assays. The sequence of SQSTM1-siRNA included 5′-GGAGUCGGAUAACUGUUCATT-′3. The sequence of negative control- (NC-) siRNA included 5′-UUCUCCGAACGUGUCACGUTT-′3.

### 2.8. Autophagic Flux Assay

Autophagic flux in RPE cells was assessed using mRFP-GFP-LC3 lentiviruses (GENECHEM, Shanghai, China). RPE cells were infected with the lentiviruses to induce the stable expression of mRFP-GFP-LC3 (lentiviral titer 1.0 × 10^8^ TU/mL, MOI = 100) and incubated for 12 h. After infection for 36 h, cells were treated accordingly. GFP^+^/mRFP^+^ (yellow) and GFP^−^/mRFP^+^ (red) puncta were detected using confocal microscopy (Leica TCS SP8).

### 2.9. Western Blot Analysis (Whole-Cell Lysis and Nuclear Lysates)

RIPA buffer (Thermo Fisher Scientific) and a Nuclear/Cytosol Extraction Kit (Beyotime, China) were used to extract total and nuclear protein according to the manufacturer's instructions. We used the following antibodies: anti-SQSTM1 antibody (CST, 39749), anti-LC3A/B antibody (CST, 12741), anti-mTOR antibody (CST, 2983), anti-phospho-mTOR antibody (CST, 5536), anti-AMPK antibody (CST, 5831), anti-phospho-AMPK antibody (CST, 50081), anti-Nrf2 antibody (Santa Cruz, sc-365949), anti-keap1 antibody (Santa Cruz, sc-514914), anti-NQO1 antibody (Santa Cruz, sc-32793), anti-HO-1 antibody (Abcam, ab52947), and anti-*β*-actin antibody (Santa Cruz, sc-47778). The blots were incubated with primary antibodies overnight at 4°C, at which point an anti-rabbit or anti-mouse secondary antibody was added for 1 h. Band intensities were quantified by ImageJ densitometry software.

### 2.10. Immunofluorescence

The cultured cells were fixed with 4% PFA for 20 min and rinsed in PBS. The fixed samples were permeabilized with 0.2% Triton X-100 for 30 min and incubated in blocking buffer (3% BSA) for 1 h at room temperature. Cultures were then stained with primary antibodies specific for Nrf2 (Servicebio, GB13148), Keap1 (Servicebio, GB118447), and SQSTM1 (Servicebio, GB1111998) overnight at 4°C. The cells were incubated with Alexa Fluor 488 or 568 (Servicebio, GB21303 or GB23303) secondary antibodies at room temperature for an hour in the dark. Nuclei were visualized by DAPI staining for another 5 min. Finally, the specific fluorescence was imaged on a fluorescence or confocal microscope.

### 2.11. H&E Staining and Immunohistochemistry

At the indicated time point after injection of NaIO_3_, freshly harvested mouse eyes were fixed overnight with eyeball-fixed liquid (R30102; Yuanye, Shanghai, China). Vertical sections (5 *μ*m thick) were deparaffinized, hydrated in gradient alcohol, and stained with hematoxylin and eosin. Pathological changes were detected by light microscopy. The thickness of the retinal layers from H&E images was measured using ImageJ software.

Endogenous peroxidase activity was eliminated by 3% H_2_O_2_. After blocking in 5% normal goat serum, the sections were incubated with primary antibodies against p-AMPK (CST, 50081), SQSTM1 (Servicebio, GB11239), Keap1 (Santa Cruz, SC-514914), and Nrf2 (Servicebio, GB13148) overnight at 4°C and biotinylated secondary antibodies (Servicebio, GB23303 or GB23301) for 1 h at room temperature. Subsequently, the sections were developed with 3,3′-diaminobenzidine (DAB), counterstained with hematoxylin, and sealed with neutral resin. The intensity of staining was determined based on the brown-yellow particles.

### 2.12. Transmission Electron Microscopy (TEM)

TEM analysis of autophagosome formation is an important method to study autophagy. After the indicated treatments, retinal tissues and RPE cells were fixed with 2.5% paraformaldehyde (pH 7.4) at 4°C overnight. Samples were dehydrated in an ethanol gradient (30%–100%) and embedded in epoxy resin. Ultrathin sections were double-stained with uranyl acetate and lead citrate. Autophagosomes were detected by TEM.

### 2.13. Detection of SOD, CAT, and MDA

After the various treatments, blood samples were centrifuged at 4000 r/min for 10 min at 4°C to collect the serum samples. In addition, RPE cells were lysed by sonication and centrifuged to collect the supernatant. Superoxide dismutase (SOD) activity, catalase (CAT) activity, and malondialdehyde (MDA) levels were determined according to the manufacturer's protocols (Nanjing Jiancheng Institute of Biotechnology, Nanjing, China).

### 2.14. Statistical Analysis

Statistical analysis was performed using SPSS 19.0 software (IBM, Armonk, NY, USA). Data are expressed as the mean ± SD and were considered to be statistically significant at *P* < 0.05 and *P* < 0.01. Two-group comparisons were analyzed using unpaired *t*-tests. For analysis with multiple comparisons, one-way analysis of variance (ANOVA) was used.

## 3. Result

### 3.1. MMDH Pill Protects the Viability of RPE Cells from NaIO_3_ Damage

**Figure 1:**
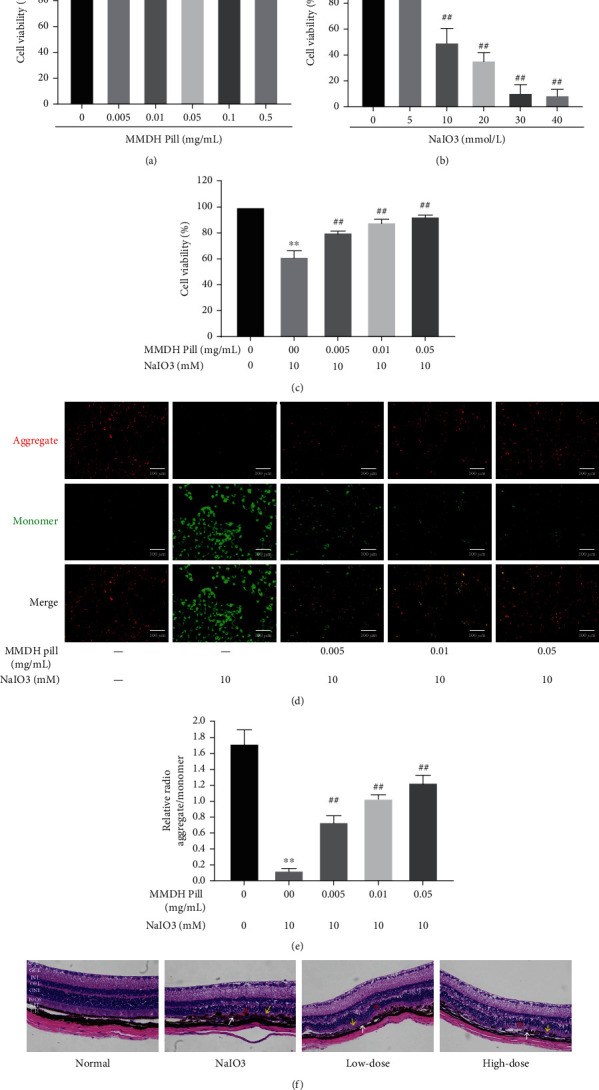
MMDH pill protected the viability of RPE cells from NaIO_3_ damage. (a, b) RPE cells were treated with MMDH pill (0, 0.005, 0.01, 0.05, 0.1, and 0.5 mg/mL) or NaIO_3_ (0, 5, 10, 20, 30, and 40 mM) for 24 h. (c) RPE cells were pretreated with MMDH pill (0-0.5 mg/mL) 24 h prior to NaIO_3_ (10 mM) treatment for 24 h. (d) Double staining of RPE cells by JC-1 is visible in the red, green, and merged channels. Bar = 100 *μ*m. (e) Quantitative assessment of ΔΨ*m*. (f) Retinal HE staining (3 mice per group). ×400 magnification. GCL: ganglion cell layer; IPL: inner plexiform layer; INL: inner nuclear layer; OPL: outer plexiform layer; ONL: outer nuclear layer; IS/OS: inner segment/outer segment; Ch: choroid; white arrow: RPE; yellow arrows: IS/OS; red arrows: ONL. The data are representative of three independent experiments and are expressed as the mean ± SD. ^∗^*P* < 0.05 and ^∗∗^*P* < 0.01 vs. the control group; ^#^*P* < 0.05 and ^##^*P* < 0.01 vs. the NaIO_3_ group.

To identify the major chemical components, MMDH pill samples were analyzed by UHPLC-MS/MS. These compounds have covered most of the main peaks in the chromatogram and different kinds of constituents were involved, such as flavonoids (quercetin, naringenin, kaempferol, luteolin, and isorhamnetin), terpenoids (ajugol, albiflorin, digoxigenin, gracillin, and pachymic acid), iridoids (loganin, sarracenin, and morroniside), and phenols (benzoic acid, gallic acid, and methyl gallate) (Supplemental Table 1 and Figure 1).

The efficacy of the MMDH pill against NaIO_3_-induced damage in RPE cells was first demonstrated. The effects of the MMDH pill and NaIO_3_ on cellular proliferation and viability were first tested by using CCK8 assays. RPE cells were treated with different concentrations of MMDH pill (0, 0.005, 0.01, 0.05, 0.1, and 0.5 mg/mL) and NaIO_3_ (0, 5, 10, 20, 30, and 40 mmol/L) for 24 h. As the drug concentration increased, MMDH pill did not impair RPE viability (Figure 1(a)) (*P* > 0.05). As shown in Figure 1(b), 10 mM NaIO_3_ treatment for 24 h reduced cell viability to approximately 50%. Based on this observation, we chose a NaIO_3_ concentration of 10 mM for the in vitro study. To investigate whether the MMDH pill could protect RPE cells from oxidative damage, cells were pretreated with MMDH pill (0.005, 0.01, and 0.05 mg/mL) for 24 hours and then treated with NaIO_3_ (10 mM) for 24 hours. Our results demonstrated that the cell viability of the NaIO_3_ group was significantly decreased compared to that of the control group, which was dose-dependently reversed by MMDH pills (Figure 1(c)). ΔΨ*m* is a fundamentally important parameter in eukaryotic cells, which represents the electric gradient across the mitochondrial inner membrane. ΔΨ*m* loss and mitochondrial outer membrane permeabilization (MOMP) are intricately linked. Apoptotic signaling downstream of cytochrome c released into the cytoplasm and caspase activation following MOMP [18]. In cells with low ΔΨ*m*, JC-1 remains in the monomers with green fluorescence. In healthy cells with high ΔΨ*m*, the accumulation of JC-1 leads to the formation of JC-1 aggregates with red fluorescence. We then used the JC-1 probe to detect MMP in each group. NaIO_3_-treated RPE cells exhibited a decreased ratio of red and green fluorescence, and the cells were undergoing apoptosis (Figures 1(d) and 1(e)). In the MMDH pill treatment group, the red fluorescence remarkably increased and the green fluorescence reduced in a dose-dependent manner (Figures 1(d) and 1(e)).

NaIO_3_ causes retinal degeneration in an AMD mouse model. As shown in Figure 1(f), HE staining showed that the retinal layer of the normal groups was regularly and evenly arranged. However, after 7 days of NaIO_3_ injection, the retina was severely damaged, the RPE layer was seen as black circular sediments, the inner segment/outer segment (IS/OS) was disordered, and the outer nuclear layer (ONL) became thinner. Compared with the NaIO_3_ group, the pretreatment of the MMDH pill reduces black circular sediments on the RPE layer and improves the change in IS/OS and ONL (Supplemental Figures 2A and 2B).

### 3.2. MMDH Pill Ameliorates Oxidative Stress and Improves Antioxidant Enzyme Activities in RPE Cells via the Keap1/Nrf2/HO-1 Signaling Pathway

**Figure 2:**
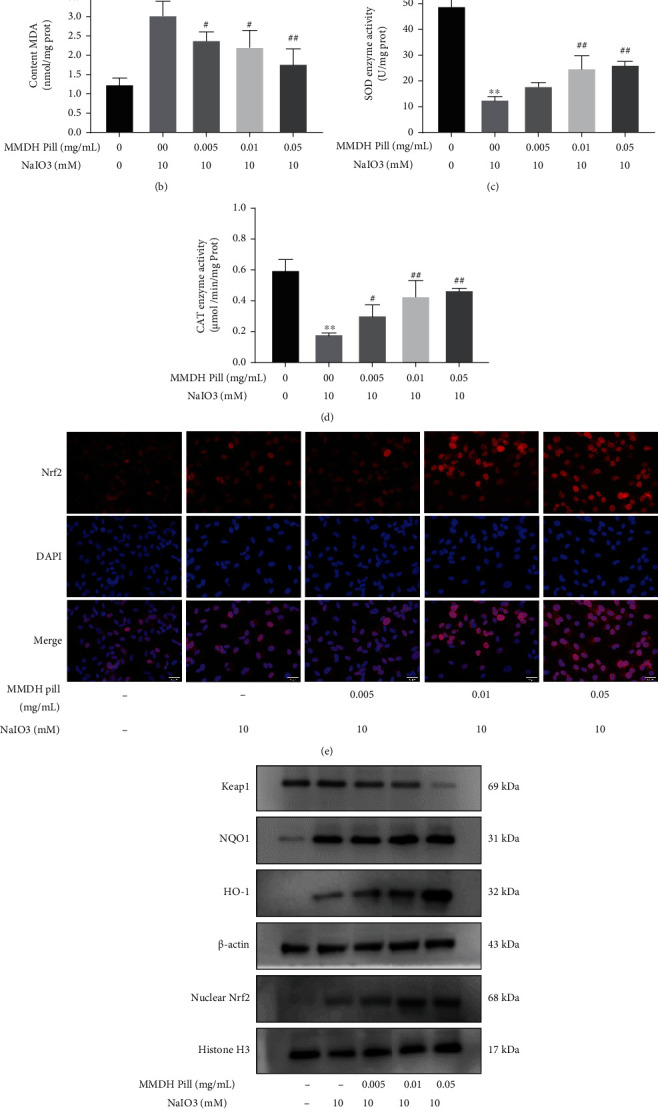
MMDH pill ameliorates oxidative stress and improves antioxidant enzyme activities in RPE cells via the Keap1/Nrf2/HO-1 signaling pathway. (a) DCFH-DA probe was used to detect the generation of ROS with fluorescence microscopy. Scale bar = 100 *μ*m. (b–d) Effect of MMDH pill pretreatment on the oxidative stress factors MDA, CAT, and SOD in RPE cells. (e) Colocalization of Nrf2 and DAPI in RPE cells was observed by immunofluorescence staining. Scale bar = 50 *μ*m. (f, g) Western blot analysis was employed to evaluate the expression of Keap1, nuclear Nrf2, NQO1, and HO-1. The data shown are representative of at least three independent experiments. ^∗^*P* < 0.05 and ^∗∗^*P* < 0.01 vs. the control group; ^#^*P* < 0.05 and ^##^*P* < 0.01 vs. the NaIO_3_ group.

ROS and MDA are the key indicators of the oxidative system. SOD and CAT are the important indicators of the antioxidative system. We investigated the generation of ROS in RPE cells treated with MMDH pill. Compared to the control cells, NaIO_3_ treatment alone for 24 h markedly increased the DCF fluorescence intensity to 4.93-fold, while its intensity in the MMDH pill (0.005, 0.01, and 0.05 mg/mL)-pretreated cells was significantly reduced (Figure 2(a)). Treatment with NaIO_3_ alone markedly increased MDA levels and rapidly reduced the activities of the antioxidant enzymes SOD and CAT, resulting in oxidative insult in RPE cells. Treatment with the MMDH pill significantly decreased the production of MDA (Figure 2(b)). The activities of SOD and CAT in the MMDH pill groups were significantly increased compared with those in the NaIO_3_ group (Figures 2(c) and 2(d)).

Nrf2 is a key transcription factor that maintains the redox balance of cells through the regulator of endogenous antioxidative enzymes [1]. To examine whether Nrf2 is activated in response to MMDH pill treatment, we investigated the intracellular localization of Nrf2 through fluorescence microscopy. Immunofluorescence results showed that Nrf2 showed weaker intensity and was predominantly localized in the cytoplasm of RPE cells in the control groups; nevertheless, MMDH pill treatment enhanced Nrf2 nuclear translocation and expression in RPE cells (Figure 2(e)). As shown in the western blot results, Keap1, nuclear Nrf2, NQO1, and HO-1 expression was increased in RPE cells exposed to NaIO_3_ for 24 h compared with control cells (Figures 2(f) and 2(g)). However, compared with NaIO_3_ treatment, MMDH pill treatment significantly increased the expression levels of nuclear Nrf2, HO-1, and NQO1 and decreased Keap1 levels (Figures 2(f) and 2(g)). Western blot analysis was consistent with the immunofluorescence staining results showing that the MMDH pill promoted cytoplasmic Nrf2 translocation into the nucleus under conditions of oxidative damage.

### 3.3. MMDH Pill Activates Nrf2/HO-1 via Autophagic KEAP1 Degradation

**Figure 3:**
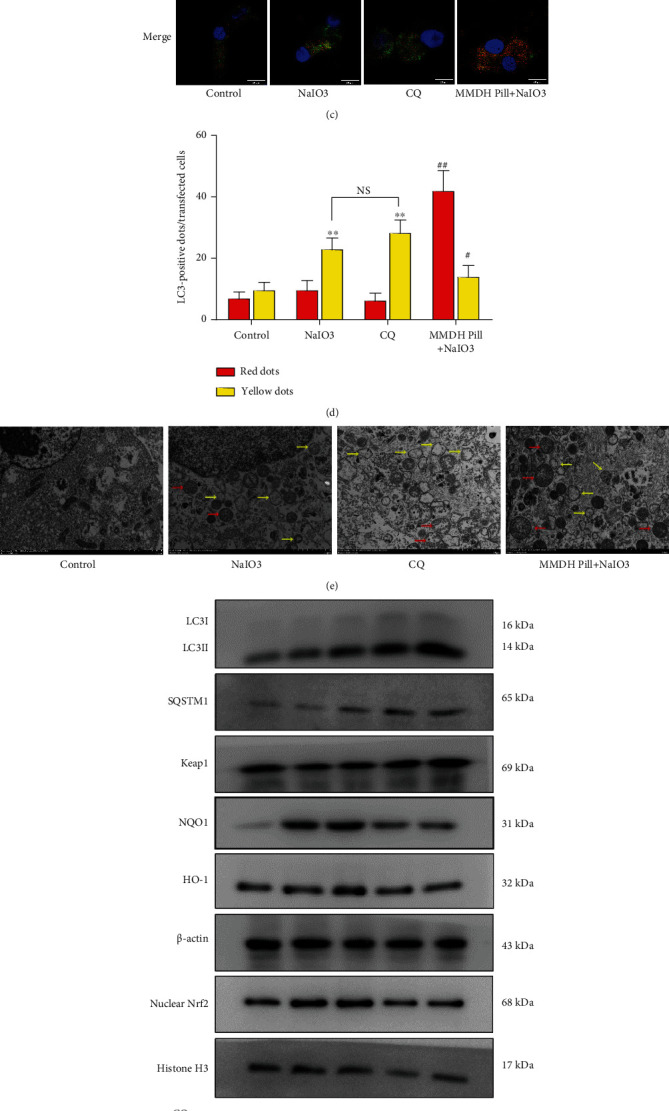
The MMDH pill activates Nrf2/HO-1 via autophagic keap1 degradation. (a, b) Immunoblot analysis of the expression of LC3II and SQSTM1 in RPE cells upon MMDH pill (0.005, 0.01, and 0.05 mg/mL) treatment for 24 h. (c, d) RPE cells were transfected with mRFP-GFP-LC3 adenovirus for 48 h. Cells treated with CQ (10 *μ*M) for 1 h, NaIO_3_ (10 mM) for 24 h, or MMDH pill (0.05 mg/mL)+NaIO_3_. The formation of autophagosomes (yellow puncta) and autolysosomes (red puncta) was detected by confocal microscopy. Scale bars = 20 *μ*m. (e) Electron microscope images of the RPE cells. Scale bar = 2 *μ*m. Yellow arrows: autophagosome; red arrows: autolysosome. (f, g) Western blot analysis of SQSTM1, LC3II, Keap1, nuclear Nrf2, NQO1, and HO-1 levels. The results shown are the means ± SDs of three independent experiments. ^∗^*P* < 0.05 and ^∗∗^*P* < 0.01 vs. the control group; ^#^*P* < 0.05 and ^##^*P* < 0.01 vs. the NaIO_3_ group; ^&^*P* < 0.05 and ^&&^*P* < 0.01 vs. the NaIO_3_ group; ^$^*P* < 0.05 and ^$$^*P* < 0.01 vs. the MMDH pill+NaIO_3_ group.

We determined autophagy by assessing the critical autophagy-related proteins LC3-II and the autophagy receptor SQSTM1. Compared with the control groups, LC3-II and SQSTM1 were increased after exposure to NaIO_3_ (Figures 3(a) and 3(b)) (*P* < 0.01), indicating autophagy induction during acute oxidative damage. Compared with the NaIO_3_ groups, the levels of LC3-II and SQSTM1 were upregulated after treatment with the MMDH pill, which showed elevated levels of autophagy. Our results showed that the levels of Nrf2, LC3-II, and SQSTM1 were significantly increased when the MMDH pill reached a higher concentration. We subsequently utilized a 0.05 mg/mL MMDH pill in our following studies. Furthermore, the protein expression levels of LC3-II and SQSTM1 were increased after MMDH pill alone treatment compared with the NaIO_3_ group, indicating that MMDH pill could enhance autophagic flux. In contrast, the Keap1 protein expression level was decreased in the MMDH pill group compared with the NaIO_3_ group (Supplemental Figures 3A–3D).

Chloroquine (CQ) is the most autophagy inhibitor that inhibits the last stage of autophagy. As can be seen from Supplemental Figure 4A, cell viability was significantly suppressed in the CQ-treated group. To monitor autophagic flux, we used a tandem fluorescently tagged LC3 lentivirus mRFP-GFP-LC3 expression system. Yellow dots (GFP^+^ and mRFP^+^) in the merged images indicate autophagosomes, and red dots (GFP^−^ and mRFP^+^) indicate the formation of autolysosomes [19]. Green dots (GFP^+^ and mRFP^−^) are more sensitive to acidic pH than RFP fluorescence and indicate defective fusion of autophagosomes with lysosomes. As shown in Figure 3(c), NaIO_3_ treatment resulted in the accumulation of yellow dots only, indicating that NaIO_3_ and CQ inhibited autophagosome-lysosome fusion in RPE cells. Treatment of RPE cells with MMDH pill enhanced red fluorescent puncta, indicating an increase in autophagic flux. TEM was also used to investigate the morphology of autophagic structures. Autophagosomes (also referred to as initial autophagic vacuoles (AVi)) have a double thin membrane compartment (7–8 nm). The sequestration membranes of autophagosomes have the distended empty space. Autolysosomes are generally delimited by a single membrane. A degradative compartment is formed by the fusion of an autophagosome or amphisome with a lysosome [20]. NaIO_3_ and CQ treatment resulted in the accumulation of autophagosomes and a decrease in autolysosomes in RPE cells. Moreover, the MMDH pill induced the production of autophagosomes and autolysosomes upon NaIO_3_ stimulation, further emphasizing the ability of MMDH pill to promote autophagic flux (Figure 3(e)).

To evaluate the necessity of autophagy and Keap1 degradation, we inhibited autophagosome-lysosome fusion with CQ in RPE cells. Immunoblot analysis revealed that MMDH pill-induced Keap1 degradation was significantly blocked in CQ-treated RPE cells (Figures 3(f) and 3(g)). In parallel with this change, the expression levels of nuclear Nrf2, HO-1, and NQO1 in the CQ-treated group were markedly decreased compared with those in the MMDH pill group. However, CQ treatment resulted in the accumulation of LC3-II and SQSTM1 proteins, suggesting a blockage of autophagic flux in RPE cells (Figures 3(f) and 3(g)). These results revealed that the MMDH pill promoted autophagic flux and autophagic Keap1 degradation in RPE cells, followed by Nrf2 activation.

### 3.4. The MMDH Pill Activates SQSTM1 in the Regulation of the KEAP1-NRF2 Pathway in response to Oxidative Stress

**Figure 4:**
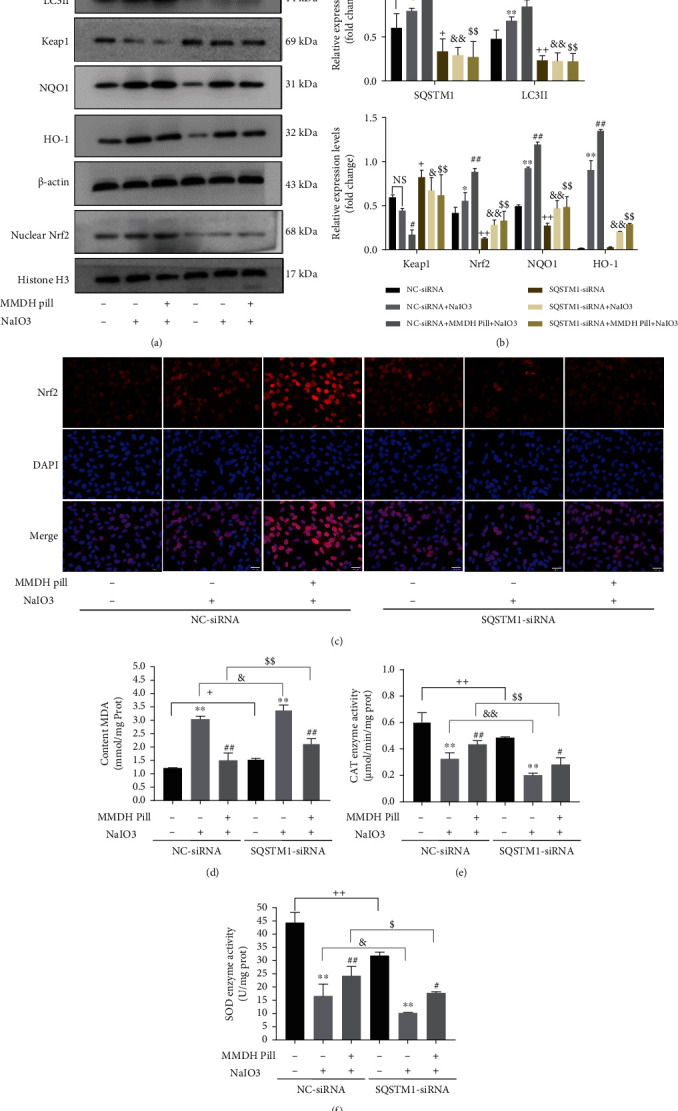
MMDH pill activates SQSTM1 in the regulation of the Keap1-Nrf2 pathway in response to oxidative stress. (a, b) RPE cells were transfected with NC-siRNA or SQSTM1-siRNA sequences. The expression of SQSTM1/Keap1/Nrf2 pathway-related proteins was evaluated using western blot analysis. (c) Colocalization of Nrf2 and DAPI in RPE cells was observed by immunofluorescence staining. Scale bar = 50 *μ*m. (d–f) The levels of MDA, CAT, and SOD were quantified in the different treatment groups. The results shown are the means ± SDs of three independent experiments. ^∗^*P* < 0.05 and ^∗∗^*P* < 0.01 vs. the NC-siRNA/SQSTM1-siRNA group; ^#^*P* < 0.05 and ^##^*P* < 0.01 vs. the NC-siRNA/SQSTM1-siRNA+NaIO_3_ group; ^+^*P* < 0.05 and ^++^*P* < 0.01 vs. the NC-siRNA group; ^&^*P* < 0.05 and ^&&^*P* < 0.01 vs. the NC-siRNA+NaIO_3_ group; ^$^*P* < 0.05 and ^$$^*P* < 0.01 vs. the NC-siRNA+MMDH pill+NaIO_3_ group.

To confirm the role of SQSTM1 in autophagic Keap1 degradation, siRNA was used to knock down SQSTM1. As can be seen from Supplemental Figure 4B, cell viability was significantly suppressed in the SQSTM1-siRNA+NaIO_3_ group. The expression of Keap1 was significantly enhanced in response to SQSTM1 knockdown compared with NC-siRNA transfected cells (Figures 4(a) and 4(b)). Similarly, siRNA knockdown of SQSTM1 markedly decreased Nrf2 nuclear translocation and the protein expression of HO-1 and NQO1 compared with NC-siRNA transfected cells (Figures 4(a)–4(c)). Our results also showed that SQSTM1 knockdown increased the levels of MDA and decreased the activities of the antioxidant enzymes SOD and CAT (Figures 4(d) and 4(e)). The MMDH pill group showed an enhancement of Keap1 degradation, while SQSTM1 siRNA reversed MMDH pill-mediated Keap1 degradation. MMDH pill treatment induced the expression of autophagy and oxidative stress proteins, including SQSTM1, LC3II, nuclear Nrf2, HO-1, and NQO1, which were dramatically downregulated by SQSTM1 knockdown (Figures 4(a) and 4(b)). MMDH pill stimulated the levels of SOD and CAT in NC-siRNA cells but not in SQSTM1-siRNA cells (Figures 4(d)–4(f)). Together, the results suggest that the MMDH pill promotes SQSTM1-dependent autophagic degradation of Keap1 and induces Nrf2 dissociation from Keap1 and translocation to the nucleus.

### 3.5. MMDH Pill Promotes SQSTM1-Mediated Autophagy Activation, Which Is AMPK/mTOR-Dependent

**Figure 5:**
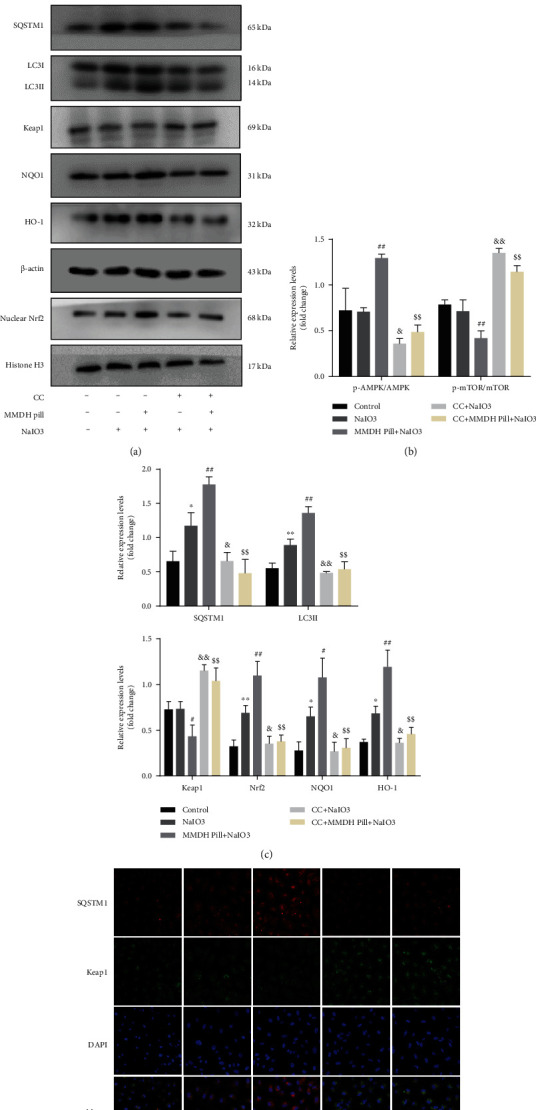
The MMDH pill promotes SQSTM1-mediated autophagy activation, which is AMPK/mTOR-dependent. (a–c) RPE cells treated with CC (10 *μ*M) for 1 h. The expression of AMPK/mTOR pathway-related proteins was evaluated using western blot analysis. (d) Representative images of SQSTM1 (red) and Keap1 (green) colocalization in RPE cells. Scale bar = 50 *μ*m. The results shown are the means ± SDs of three independent experiments. ^∗^*P* < 0.05 and ^∗∗^*P* < 0.01 vs. the control group; ^#^*P* < 0.05 and ^##^*P* < 0.01 vs. the NaIO_3_ group; ^&^*P* < 0.05 and ^&&^*P* < 0.01 vs. the NaIO_3_ group; ^$^*P* < 0.05 and ^$$^*P* < 0.01 vs. the MMDH pill+NaIO_3_ group.

Although our results demonstrated that SQSTM1 played a key role in Keap1 degeneration, the mechanism of upregulated SQSTM1 expression was not known. AMPK/mTOR is among the most common pathways in the process of autophagy [21, 22]. To characterize the potential mechanisms by which MMDH pill induced autophagy, we examined the signaling pathways regulated by MMDH pill treatment. The MMDH pill alone treatment significantly increased AMPK phosphorylation and decreased mTOR phosphorylation compared with the NaIO_3_ group (Supplemental Figures 3A and 3B). We pretreated RPE cells with 10 *μ*M dorsomorphin (Compound C (CC)), an AMPK inhibitor, for 1 h to assess the effect of AMPK-MTOR signaling on autophagy and oxidative stress. As can be seen from Supplemental Figure 4C, cell viability was significantly suppressed in the CC-treated group. CC pretreatment significantly reduced the ratio of p-AMPK to AMPK, as well as the expression of SQSTM1, LC3II, nuclear Nrf2, HO-1, and NQO1, compared to the NaIO_3_ group. The ratio of p-mTOR to mTOR and Keap1 was increased after CC treatment (Figure 5(a)). MMDH pill-induced increases in p-AMPK, SQSTM1, LC3II, and Nrf2 target protein levels were significantly suppressed in CC-treated RPE cells. Consistently, the MMDH pill mediated autophagic KEAP1 degradation, which was blocked in CC-treated RPE cells.

We also studied the colocalization of SQSTM and Keap1 by confocal immunofluorescence analysis. MMDH pill treatment induced less colocalization of Keap1 and SQSTM1 in RPE cells than CC+MMDH pill+NaIO_3_. Together, these findings suggest that the MMDH pill can activate SQSTM1-induced autophagy activation dependent on AMPK phosphorylation (Figure 5(a)).

### 3.6. MMDH Pill Protects Retinal Tissue in NaIO_3_-Induced Retinal Degeneration

**Figure 6:**
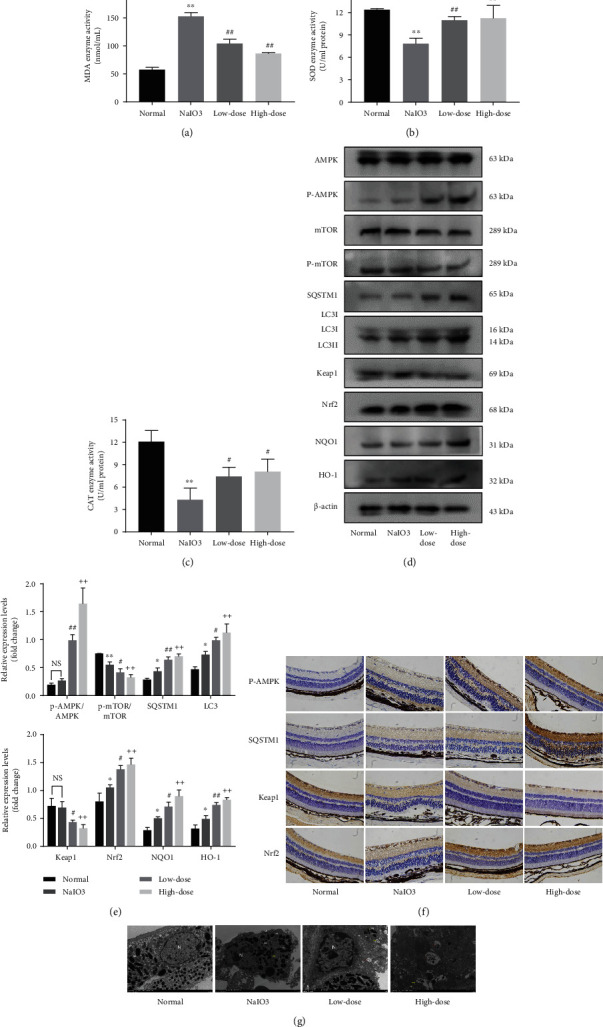
MMDH pill protects retina tissue in NaIO_3_-induced retinal degeneration. (a–c) Effect of MMDH pill on the activities of SOD and CAT, as well as MDA levels of mouse serum (5 mice per group). (d, e) Western blot analysis of AMPK/mTOR pathway-related proteins in different treatment groups (5 mice per group). The results shown are the means ± SDs of three independent experiments. ^∗^*P* < 0.05 and ^∗∗^*P* < 0.01 vs. the normal group; ^#^*P* < 0.05 and ^##^*P* < 0.01 vs. the NaIO_3_ group; ^+^*P* < 0.05 and ^++^*P* < 0.01 vs. the NaIO_3_ group. (f) Representative images of positive staining for p-AMPK, SQSTM1, Keap1, and Nrf2 in rat retinal tissues by immunohistochemistry (4 mice per group). ×400 magnification. (g) Electron microscopy images of the retinal ultrastructure (3 mice per group). Scale bar = 2 *μ*m. N: nucleus; Mi: examples of mitochondria; yellow arrows: autophagosome; red arrows: autolysosome.

As in vitro studies have shown that MMDH pills promote autophagy via the AMPK pathway, we next investigated the efficacy of MMDH pills in an in vivo AMD model. To assess the impact of MMDH pill treatment on NaIO_3_-induced oxidative damage, we measured the serum antioxidant capacity. Acute NaIO_3_ injection resulted in significant increases in serum MDA and significantly decreased serum SOD and CAT (Figures 6(a)–6(c)). However, with 0.22 and 0.44 g/mL/day MMDH pill oral pretreatment, there were significant decreases in the serum MDA, while there were significant increases in the serum SOD concentrations and CAT concentrations when compared to the NaIO_3_-induced mouse model (Figures 6(a)–6(c)).

To evaluate the protective effects of the MMDH pill, western blot analysis revealed a significantly increased P-AMPK/AMPK ratio, SQSTM1, LC3II, Nrf2, NQO1, and HO-1 in the MMDH pill-pretreated animals (0.44 g/mL/d) compared with the NaIO_3_ mice (Figure 6(d)). Together with this, a reduction in the p-mTOR/mTOR ratio and Keap1 was observed in the MMDH pill-pretreated animals. Furthermore, we used immunohistochemistry staining of retinal tissues. The expression of p-AMPK, SQSTM1, and Nrf2 in the retina showed weak positive staining (brown staining) in the NaIO_3_ group. However, compared to the NaIO_3_ group, pretreatment with the MMDH pill significantly increased the expression of p-AMPK, SQSTM1, and Nrf2 and reduced the expression of Keap1 in the ONL, INL, and RPE layer (Figure 6(f)). To further determine whether autophagy increased, TEM was used to assess the number of autophagosomal–lysosomal cells in these retinas. As shown in Figure 6(g), the normal group contained healthy mitochondria, nuclei, and pigment particles. In NaIO_3_-treated animals, there was obvious mitochondrial swelling or cristae disruption and the accumulation of autophagosomes. In MMDH pill-treated animals, newly formed and mature autophagosomal–lysosomal structures could be detected.

## 4. Discussion

Phenotypically, advanced AMD can be divided into two basic forms: neovascular (wet) and atrophic (dry). Dry AMD may progress to geographic atrophy (GA) associated with the loss of RPE and photoreceptors. Currently, no effective treatments for dry forms or GA forms of the disease exist. Supplementation with vitamin C, vitamin E, beta carotene, and zinc with dry AMD has been demonstrated to reduce the risk of progression to advanced AMD [23]. We previously demonstrated that TCM achieved higher visual acuity than vitamin C and E in dry AMD [24]. Oxidative stress ultimately involves an excess of ROS that contribute to protein misfolding and aggregation and evoke RPE dysfunction [25]. Several studies have identified Nrf2 as an essential signaling system in RPE degeneration in dry AMD [26, 27]. Keap1 is a negative regulator of Nrf2 activation via direct binding to Nrf2, which can lead to Nrf2 degradation in the resting state. Here, we observed that MMDH pill prevents NaIO_3_-induced RPE cell and animal model oxidative damage, which was dependent on the Keap1/Nrf2 pathway (Figures 2(a)–2(g) and Figures 6(a)–6(d)). However, cell viability in the MMDH pill group (0.1 mg/mL and 0.5 mg/mL) was not significantly increased compared to that in the NaIO_3_ group. The possible reason is that high concentrations of MMDH pills and NaIO_3_ treatment changed the osmotic stresses of RPE cells. Cell viability was inversely correlated with hyperosmotic stresses. Consistent with these results, MMDH pill induced the expression of phase 2 antioxidant genes, such as NQO1, HO-1, SOD, and CAT, and decreased ROS contents in RPE cells.

Autophagy is a homeostatic mechanism that recycles damaged proteins and other intracellular material by delivering them in double membrane vesicles for lysosomal degradation [28]. Recently, related studies have reported that disruption of autophagic degradation in RPE leads to the accumulation of damaged organelles, extracellular drusen deposits, and lipofuscin contributes to the pathogenesis of AMD [29]. Increased autophagy capacity can also defend against NaIO_3_-induced oxidative stress, thereby increasing the survival of RPE cells [30]. Our results show that MMDH pill treatment could effectively promote the development of autophagic flux (Figures 3(a)–3(e) and Supplemental Figures 3A–3D). As shown in Figure 3(f), the autophagy inhibitor CQ increased autophagosome accumulation and suppressed MMDH pill-induced Keap1 degeneration. MMDH pill regulated the Keap1-Nrf2 pathway to protect against NaIO_3_-induced oxidative stress in an autophagy-dependent manner (Figure 3(f) and Figures 6(d)–6(f)). As MMDH pill upregulated the expression of SQSTM1 in RPE cells and mouse models, we further studied this mechanism to defend against the progression of oxidative stress. SQSTM1 is an autophagy receptor protein that promotes aggregate protein clearance and is associated with major neurodegenerative disorders [31]. SQSTM1 has been used as an indicator of autophagic flux. A recent study showed that increased SQSTM1 expression could activate autophagy [32]. Increased SQSTM1 levels may result from transcriptional upregulation or indicate the possible inhibition of autophagosome degradation [20]. Several studies have shown that SQSTM1 promotes autophagic degradation of Keap1 against oxidative stress [13, 33]. Therefore, we hypothesized that MMDH pill promoted Nrf2 nuclear translocation through enhanced SQSTM1. Conventional autophagy, evaluated by measuring SQSTM1 and LC3-II protein levels, was decreased significantly in RPE cells with SQSTM1 knockdown. Furthermore, SQSTM1 knockdown significantly increased Keap1 and MDA levels and decreased nuclear Nrf2, NQO1, HO-1, SOD, and CAT levels (Figures 4(a)–4(f)). However, in RPE cells with SQSTM1 siRNA, MMDH pill failed to increase SQSTM1, LC3II, Nrf2 nuclear translocation, and Keap1 degeneration, which indicated that disrupted SQSTM1 might be associated with Nrf2 nuclear translocation (Figures 4(a)–4(f)). Based on these results, we suggest that SQSTM1 could be a key regulator of the Keap1/Nrf2 pathway, as well as autophagy. The molecular mechanism underlying SQSTM1-dependent activation was not clearly defined.

Numerous studies have shown that promoting autophagy could reduce the progression of AMD through various signaling pathways, such as the PI3K/protein kinase B (Akt)/mTOR pathway [34], the AMPK/mTOR pathway [35], and the SQSTM1/Keap1/Nrf2 pathway [36]. A recent study demonstrated that berberine enhances autophagy in RPE cells via the activation of the AMPK pathway and that H_2_O_2_-induced oxidative damage relies on autophagy [37]. Phosphorylation of AMPK inhibits the activity of mTOR and then induces autophagy [38]. Wang et al. found that cordycepin prevents radiation ulcers by inhibiting cell senescence via SQSTM1-Keap1-NRF2 and AMPK in rodents [39]. Lu et al. found that Adhatoda vasica Nees. relieves *tert*-butyl hydroperoxide-induced oxidative stress and activates the AMPK/p62/Nrf2 pathway [40]. Consistent with these findings, we demonstrated that the MMDH pill enhances AMPK phosphorylation, decreases mTOR phosphorylation, and promotes the interaction between SQSTM1 and Keap1 (Figures 5(a)–5(d) and Figures 6(d)–6(f)). CC, a well-known AMPK inhibitor, was preadded, and we found significantly decreased AMPK phosphorylation and SQSTM1 levels in all CC-treated groups (Figures 5(a)–5(d)). We found that the MMDH pill could activate autophagy through the phosphorylation of AMPK, which is followed by Keap1 degradation in response to oxidative stress.

## 5. Conclusion

In conclusion, our in vitro and in vivo experiments showed that the MMDH pill initiates autophagy by activating AMPK/SQSTM1/Keap1 signaling and promotes autophagic flux. Although additional investigation is still needed, the present results may offer basic information about the antioxidative effect of the MMDH pill.

## Data Availability

The original contributions presented in the study are included in the article. Further inquiries can be directed to the corresponding author.

## Abbreviations

AMD: Age-related macular degeneration
RPE: Retinal pigment epithelium
MMDH pill: Ming-Mu-Di-Huang-Pill
ROS: Reactive oxygen species
CQ: Chloroquine
CC: Compound C
SQSTM1/p62: Sequestosome-1
Keap1: Kelch-like ECH-associated protein 1
Nrf2: Nuclear factor (erythroid-derived 2)-like 2
HO-1: Heme oxygenase-1
NQO1: Nicotinamide adenine dinucleotide phosphate quinone dehydrogenase 1
LC3: Microtubule-associated protein 1 light chain 3 (MAP1LC3)
AMPK: Adenosine monophosphate-activated protein kinase
mTOR: Mechanistic target of rapamycin
SOD: Superoxide dismutase
CAT: Catalase
MDA: Malondialdehyde.

## Supplementary Materials

Supplementary 1 Supplemental Figure 1: base peak ion (BPI) chromatogram of MMDH pill.

Supplementary 2 Supplemental Figure 2: the thickness of ONL and the number of deposits on the RPE layer.

Supplementary 3 Supplemental Figure 3: MMDH pill promotes autophagy in RPE cells.

Supplementary 4 Supplemental Figure 4: (a) viability of cells treated with CQ; (b) viability of cells with SQSTM1 knockdown; (c) viability of cells treated with CC.

Supplementary 5 Supplemental Table 1: the detailed information of identified components in positive ion mode and negative ion mode by UHPLC-ESI-Q-TOF-MS/MS.
