# Supplementary figures and images for: Ming-Mu-Di-Huang-Pill Activates SQSTM1 via AMPK-Mediated Autophagic KEAP1 Degradation and Protects RPE Cells from Oxidative Damage
Source: Oxid Med Cell Longev. 2022 Mar 25;2022:5851315. doi: 10.1155/2022/5851315 (PMC8976466; doi:10.1155/2022/5851315)

**A**


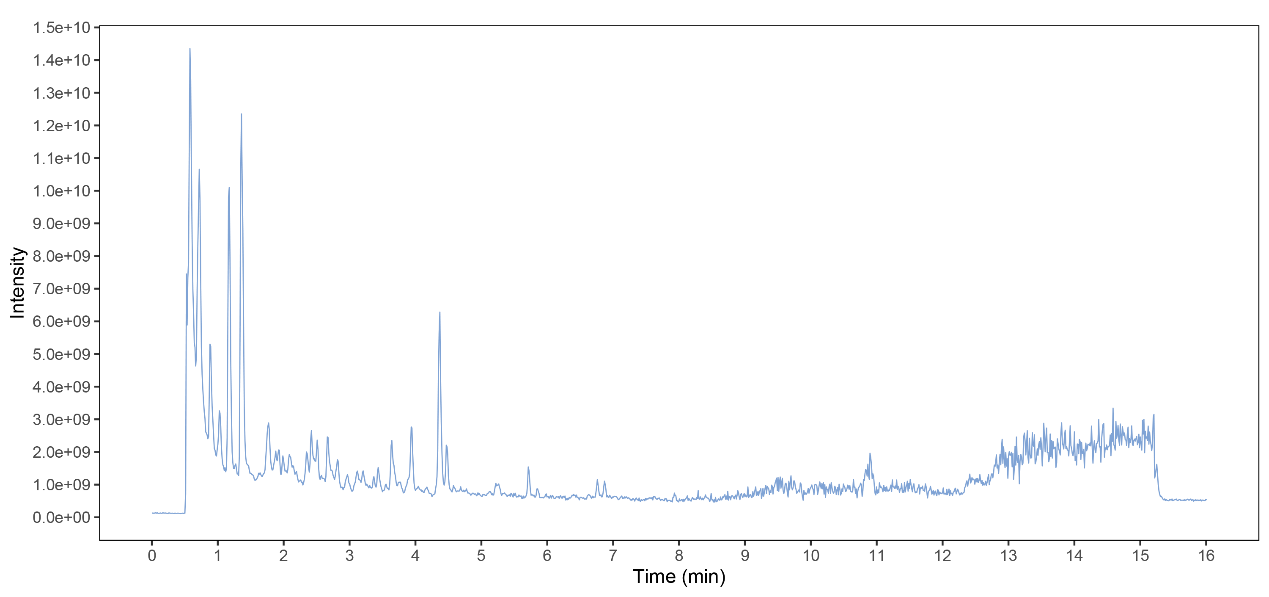


**B**


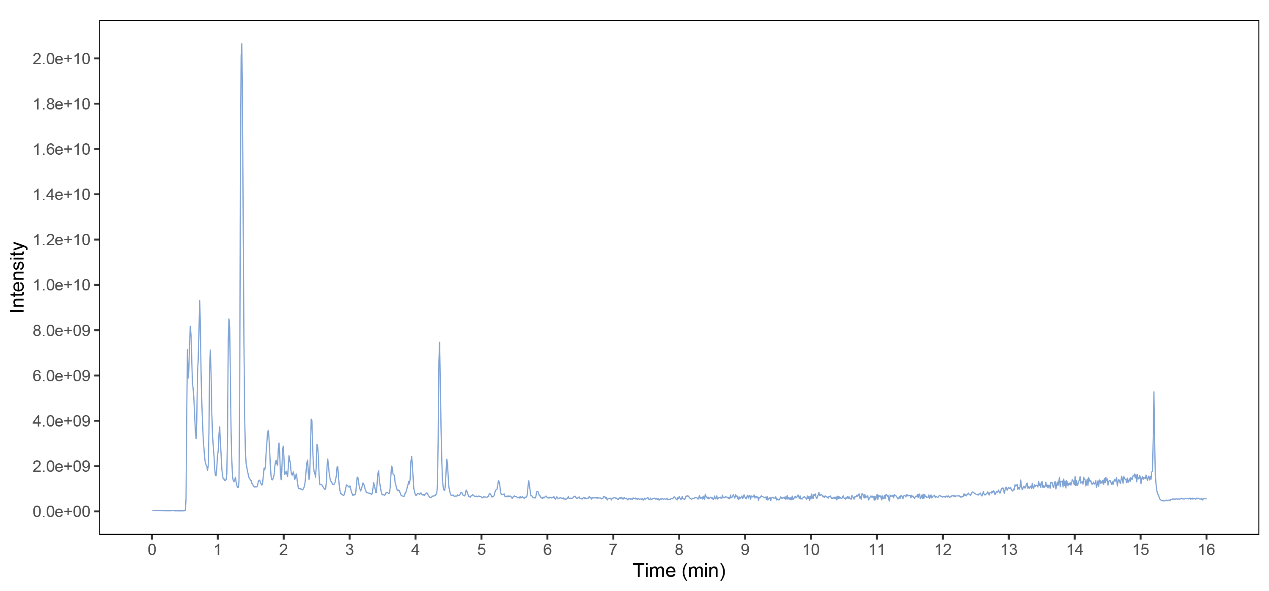

Supplement: Supplementary 1 — Supplemental Figure 1: base peak ion (BPI) chromatogram of MMDH pill. [file 5851315.f1.doc]
