# Supplementary material for: Ming-Mu-Di-Huang-Pill Activates SQSTM1 via AMPK-Mediated Autophagic KEAP1 Degradation and Protects RPE Cells from Oxidative Damage
Source: Oxid Med Cell Longev. 2022 Mar 25;2022:5851315. doi: 10.1155/2022/5851315 (PMC8976466; doi:10.1155/2022/5851315)
Supplement: Supplementary 2 — Supplemental Figure 2: the thickness of ONL and the number of deposits on the RPE layer. [file 5851315.f2.doc]

A

**120**

**ONL thickness (% of normal)**

**100**

**80**

**#**

**++**

**60**

******

**40**

**20**

**Normal**

**NaIO3**

**Low-dose**

**High-dose**

**0**

**B**

**Normal**

**NaIO3**

**Low-dose**

**High-dose**

**0**

**3**

**6**

**9**

**12**

**15**

******

**#**

**++**

**Number of deposits**
