# Supplementary material for: Ming-Mu-Di-Huang-Pill Activates SQSTM1 via AMPK-Mediated Autophagic KEAP1 Degradation and Protects RPE Cells from Oxidative Damage
Source: Oxid Med Cell Longev. 2022 Mar 25;2022:5851315. doi: 10.1155/2022/5851315 (PMC8976466; doi:10.1155/2022/5851315)
Supplement: Supplementary 3 — Supplemental Figure 3: MMDH pill promotes autophagy in RPE cells. [file 5851315.f3.doc]

**
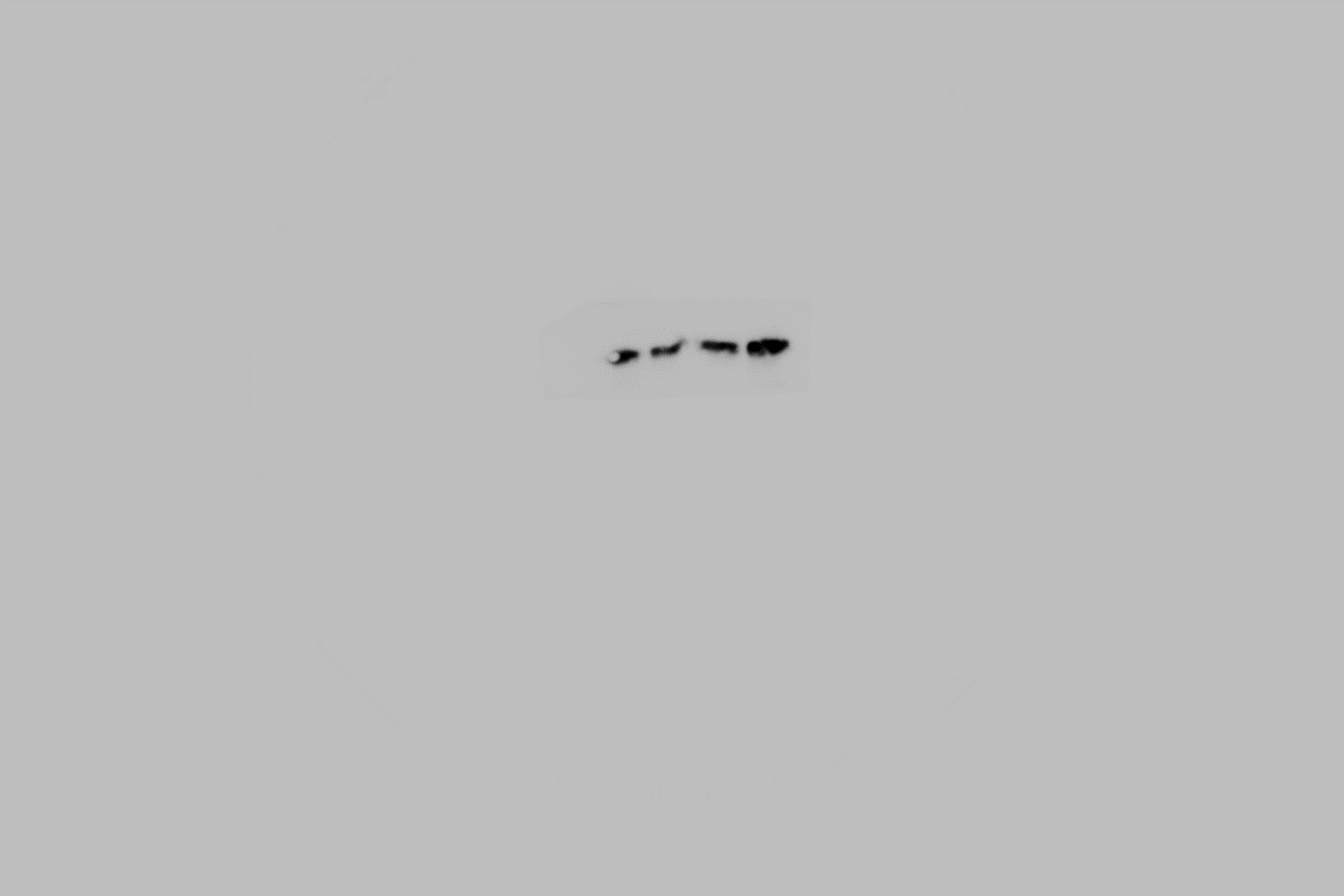

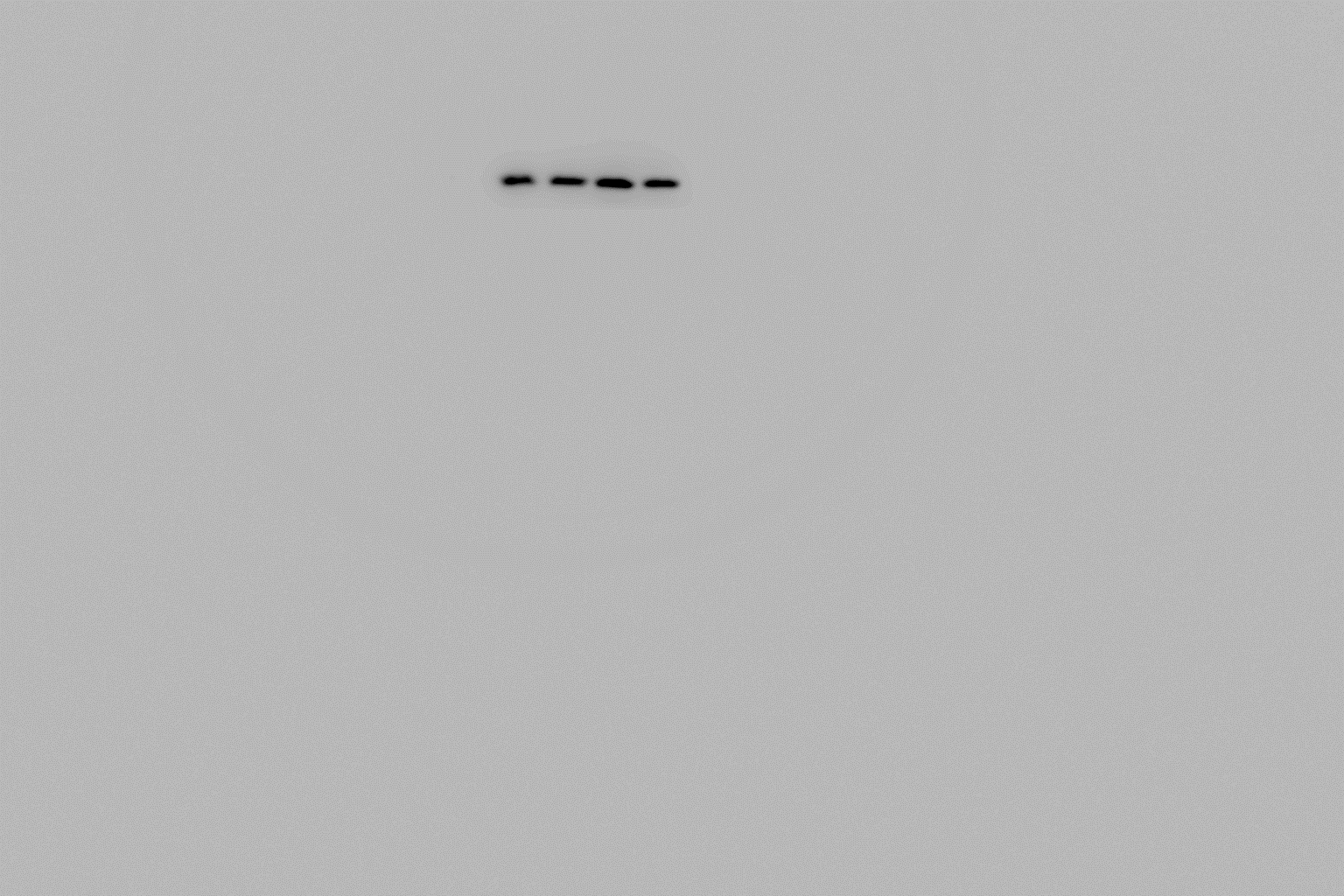

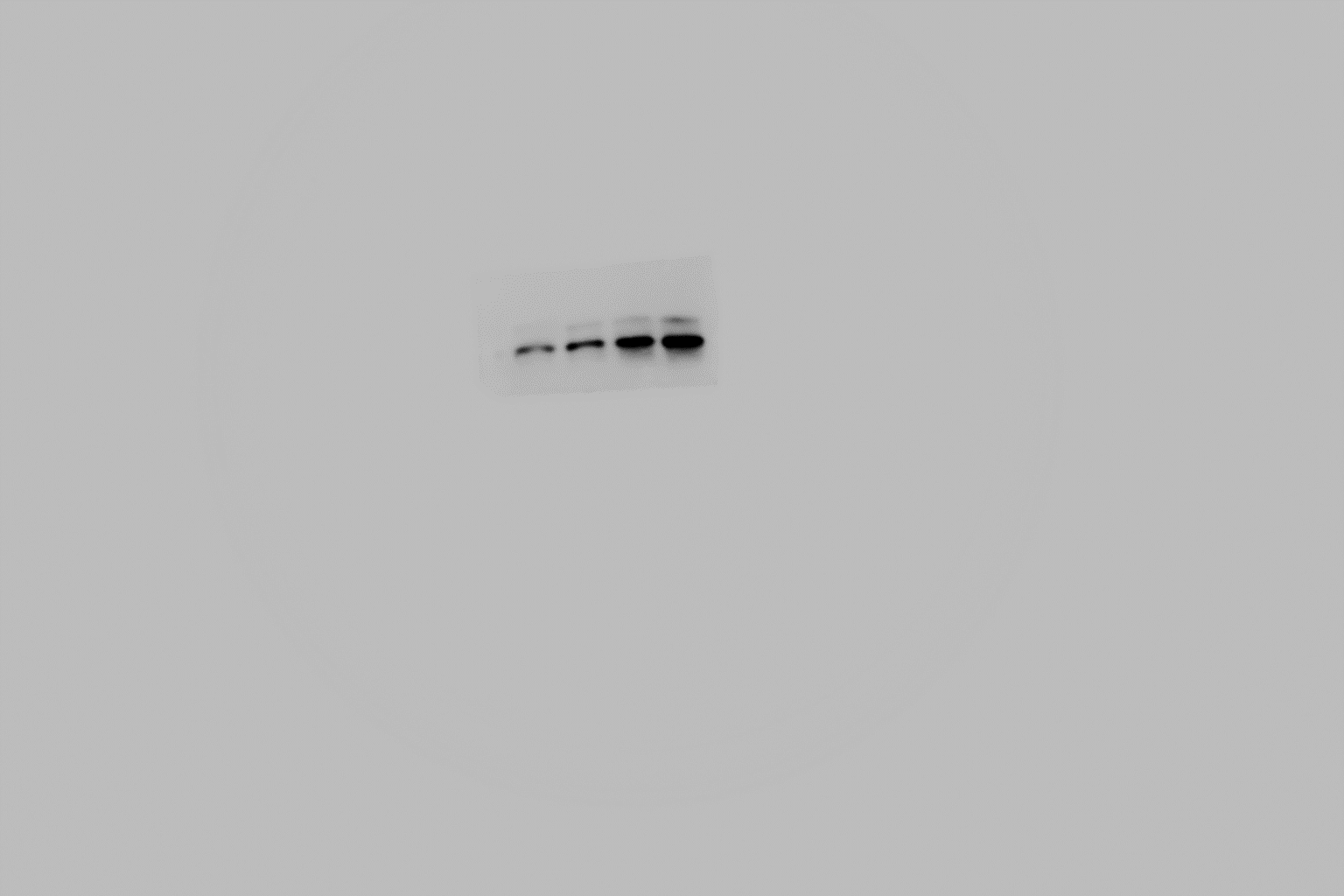

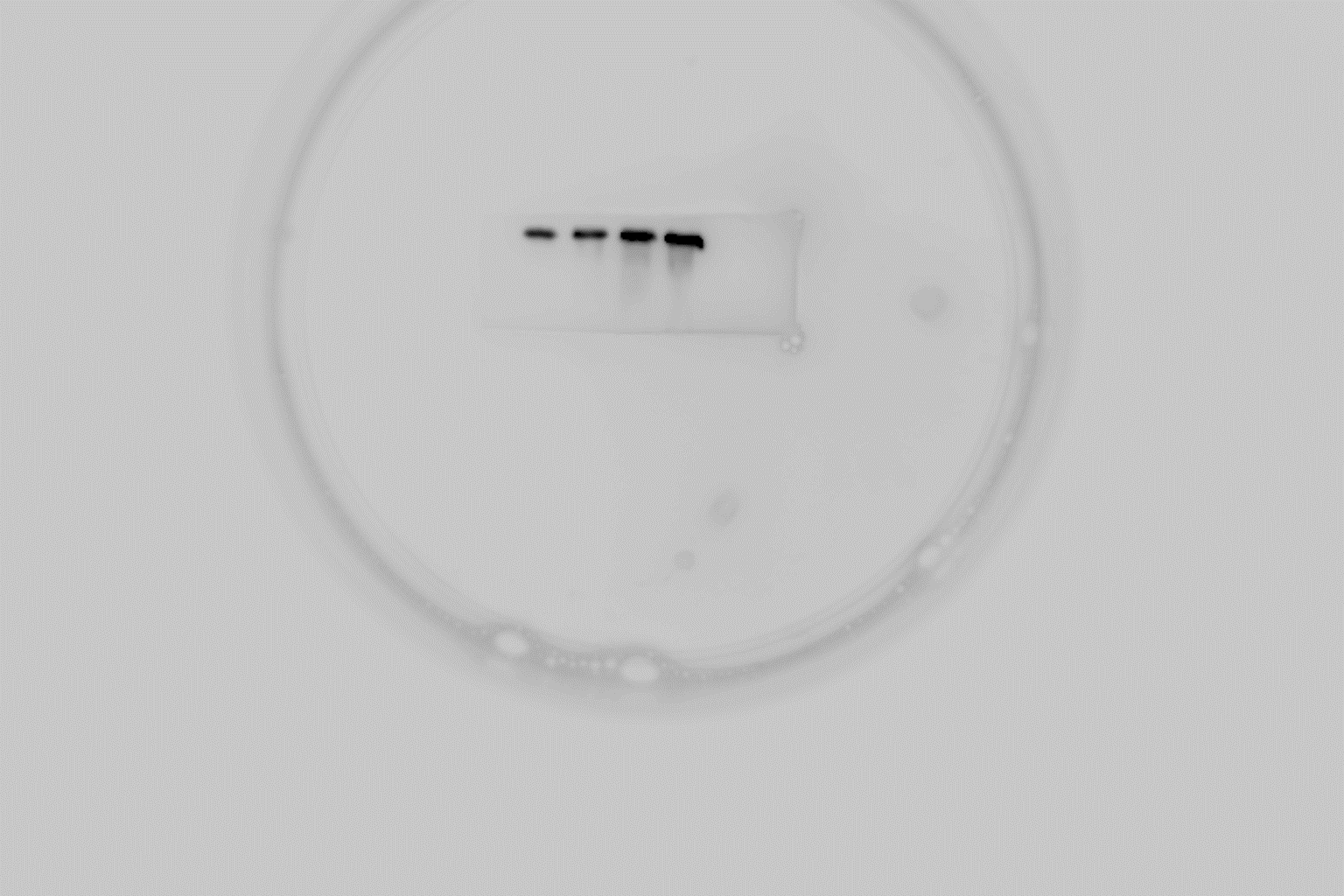

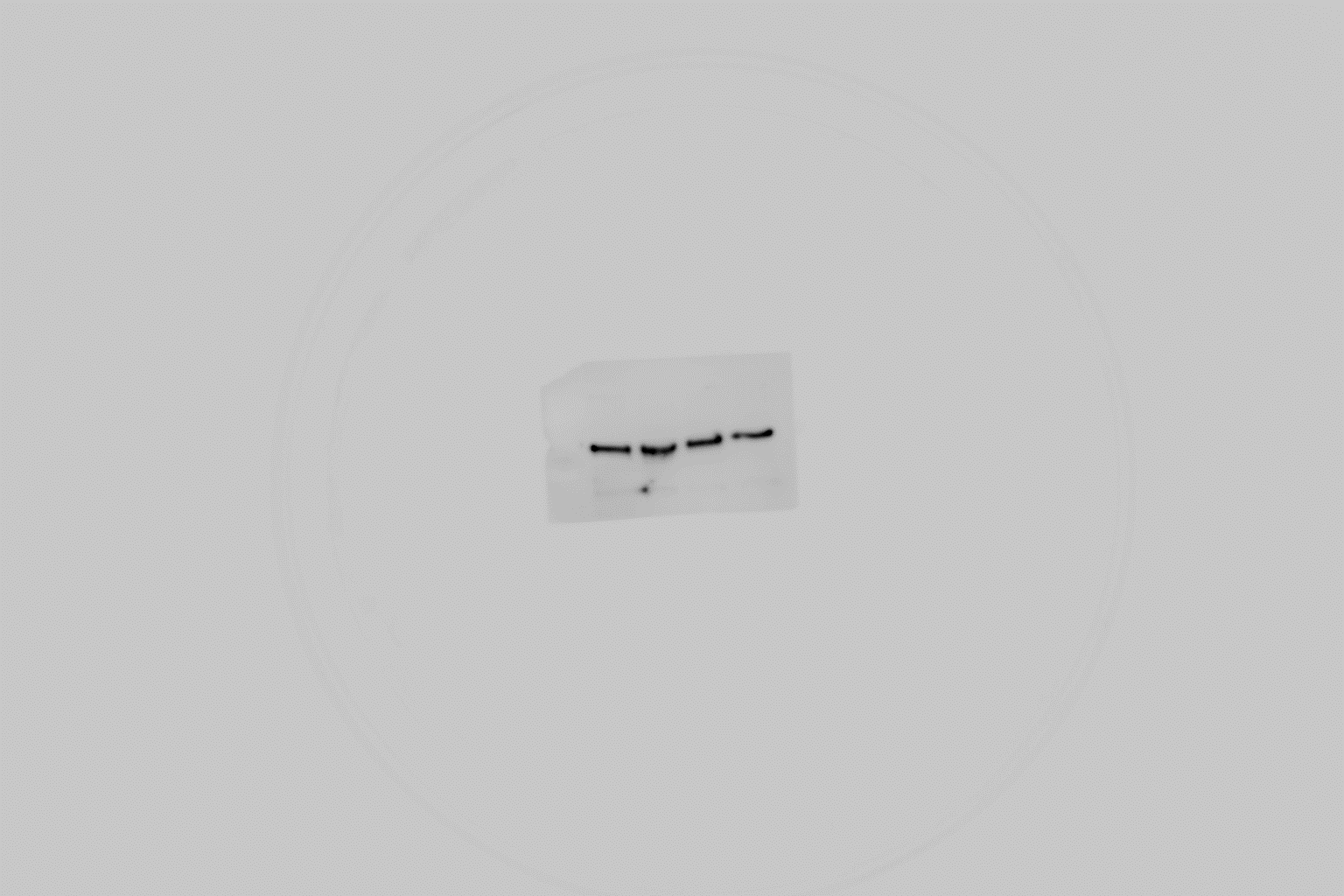

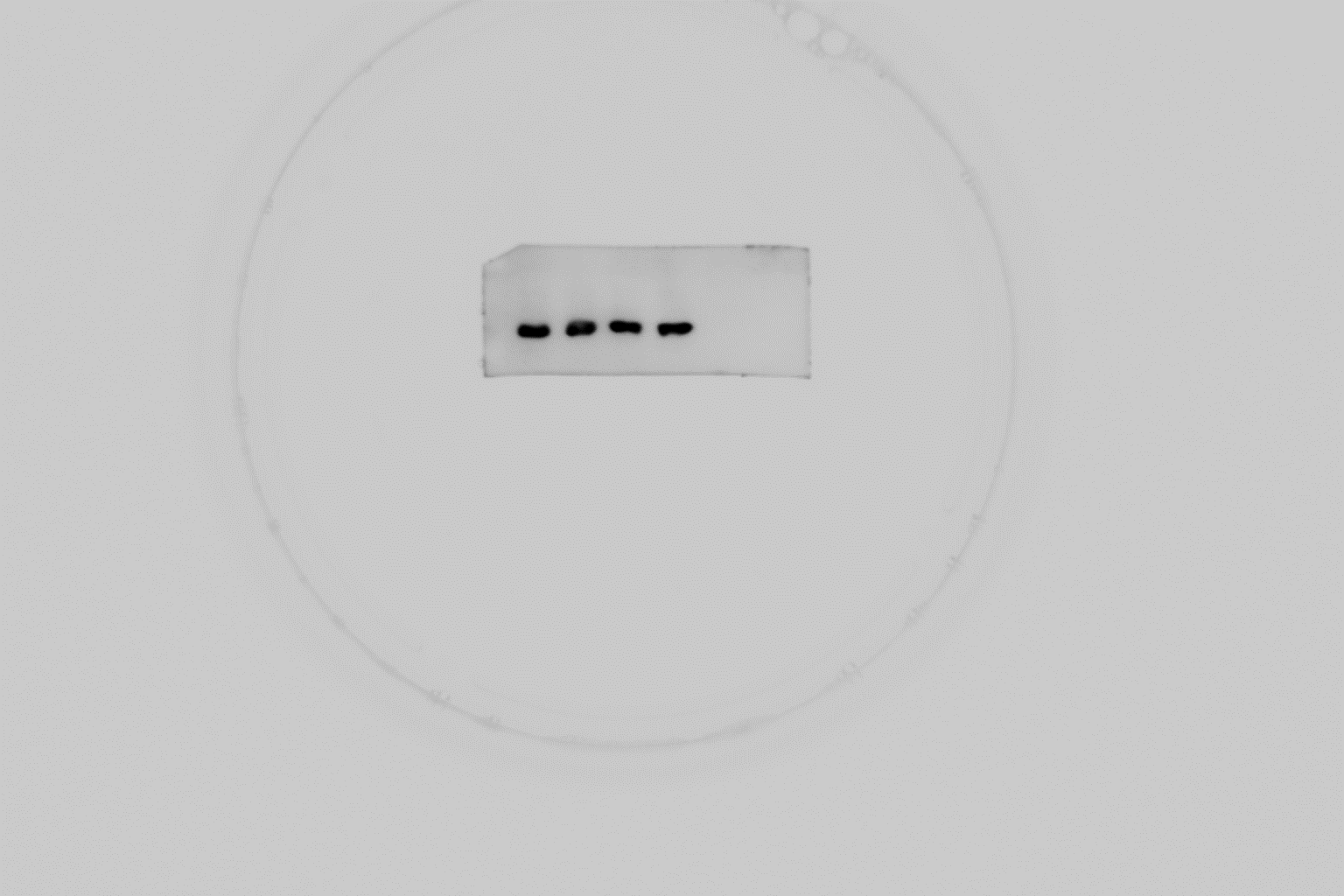

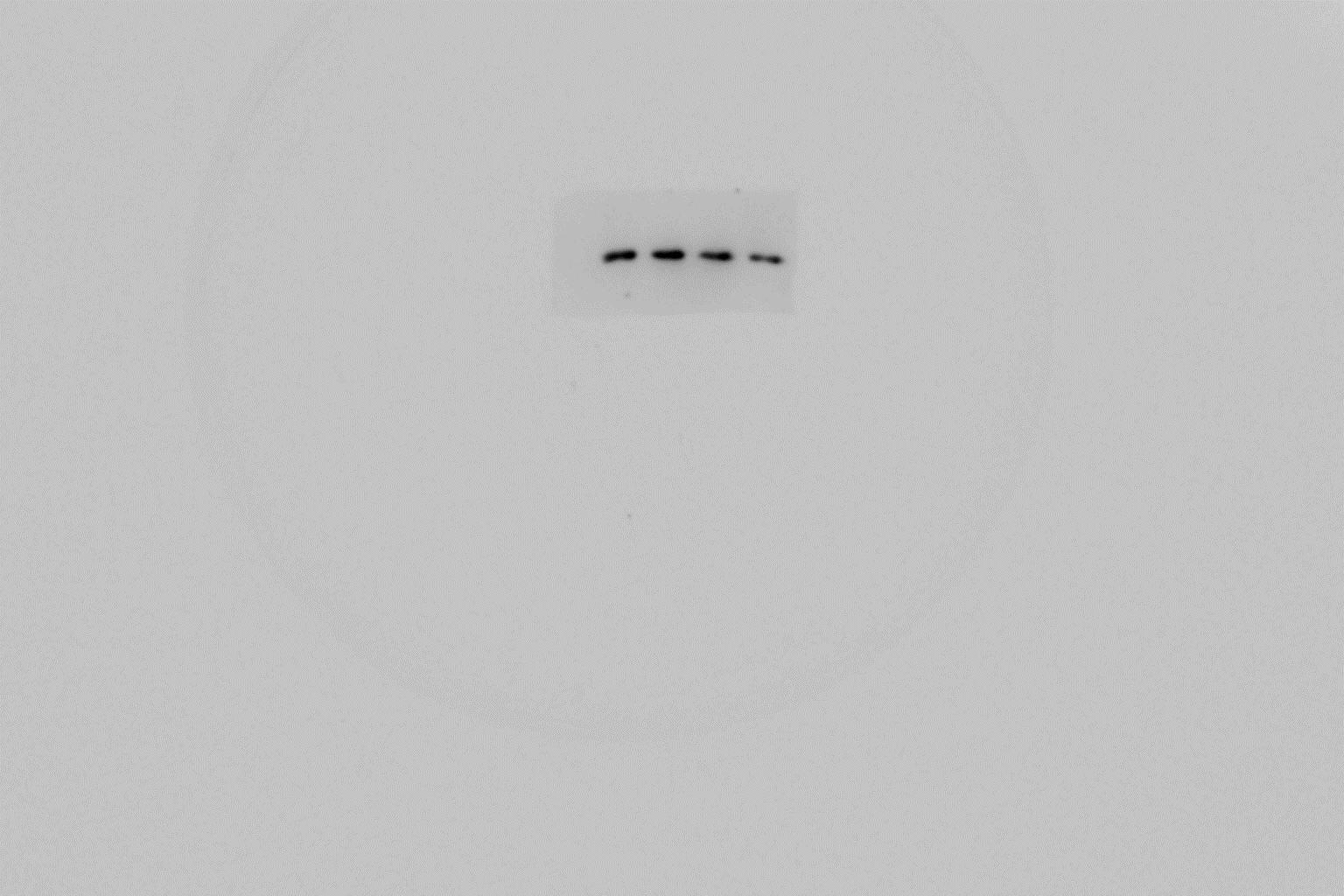

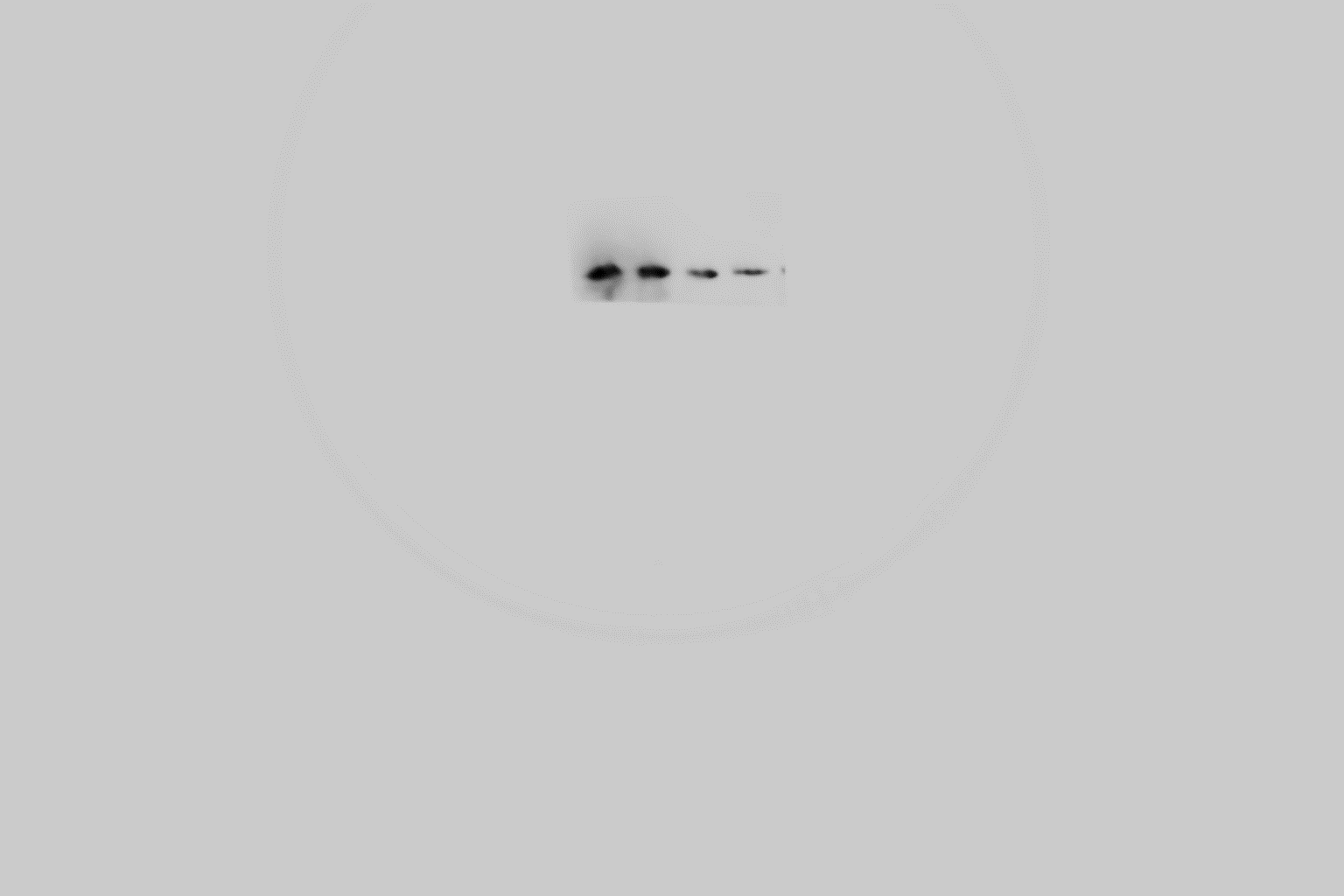
A**

**_**

**_**

**+**

**+**

**+**

**_**

**_**

**β-actin**

**AMPK**

**63 kDa**

**P-AMPK**

**63 kDa**

**mTOR**

**289 kDa**

**P-mTOR**

**289 kDa**

**LC3I**

**16 kDa**

**14 kDa**

**LC3II**

**SQSTM1**

**65 kDa**

**Keap1**

**69 kDa**

**43 kDa**

**MMDH pill（mg/mL）**

- **— + +**

**NaIO3（mM）**

- **+ — +**

**B**

**2.0**

**Relative expression levels**

**(fold change)**

**Control**

**&&**

**1.5**

**NaIO3**

**#**

**NS**

**MMDH pill**

**1.0**

**MMDH pill+NaIO3**

**##**

**&&**

**0.5**

**0.0**

**p-AMPK/**

**AMPK**

**p-mTOR/**

**mTOR**

**1.5**

**Relative expression levels**

**(fold change)**

**&**

NS

**1.0**

**##**

**##**

**&&**

**##**

**0.5**

*

*

**&&**

**0.0**

**SQSTM1**

**LC3II**

**Keap1**


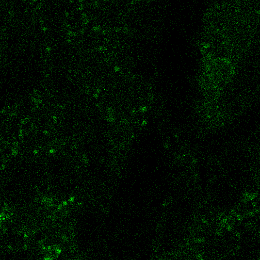

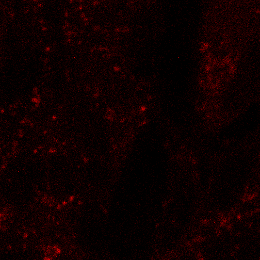

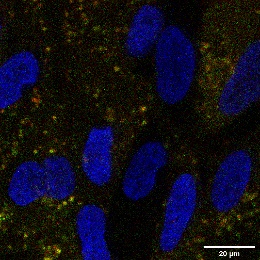

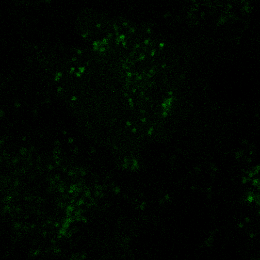

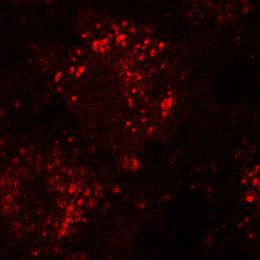

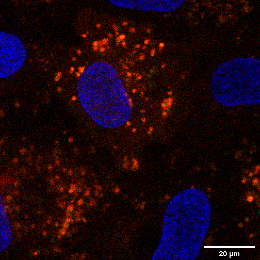

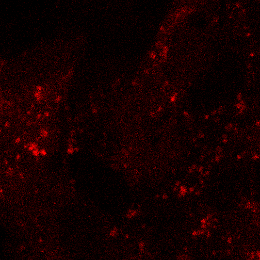
C


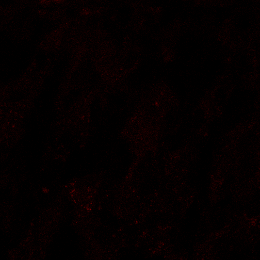


**mRFP-LC3**


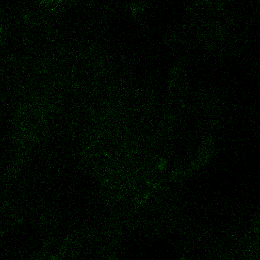

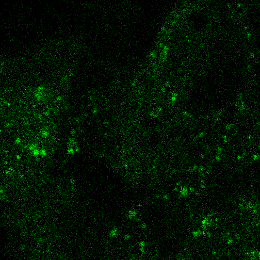


**GFP-LC3**


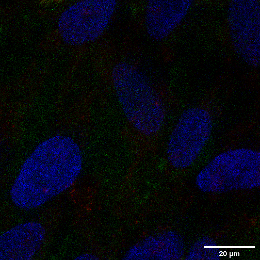

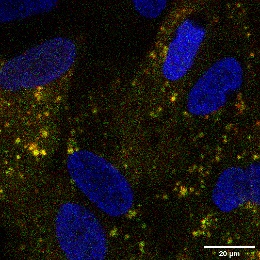


**Merge**

**Control**

**NaIO3**

**MMDH pill+**

**NaIO3**

**MMDH pill**

**Merge**

**D**

**Control**

**NaIO3**

**MMDH Pill**

**MMDH Pill**

**+NaIO3**

**0**

**20**

**40**

**60**

**80**

red dots

yellow dots

******

**&&**

**#**

**##**

**&&**

**LC3-positive dots/transfected cells**
