# Supplementary material for: Ming-Mu-Di-Huang-Pill Activates SQSTM1 via AMPK-Mediated Autophagic KEAP1 Degradation and Protects RPE Cells from Oxidative Damage
Source: Oxid Med Cell Longev. 2022 Mar 25;2022:5851315. doi: 10.1155/2022/5851315 (PMC8976466; doi:10.1155/2022/5851315)
Supplement: Supplementary 4 — Supplemental Figure 4: (a) viability of cells treated with CQ; (b) viability of cells with SQSTM1 knockdown; (c) viability of cells treated with CC. [file 5851315.f4.doc]

**A**

**120**

**100**

**##**

**80**

**60**

******

**$$**

**40**

**&&**

**20**

**Control**

**0**

**NaIO3**

**MMDH pill**

**+NaIO3**

**CQ+NaIO3**

**CQ+MMDH**

**Pill+NaIO3**

**B**

**120**

**NS**

**100**

**##**

**Cell viability(%)**

**80**

**60**

**

**$$**

**40**

**&&**

**20**

**0**

**— — + — — +**

**— + + — + +**

**MMDH Pill**

**NaIO3**

**NC-siRNA**

**SQSTM1-siRNA**

**C**

**120**

**100**

**##**

**80**

**60**

******

**$$**

**40**

**&&**

**20**

**0**

**Control**

**NaIO3**

**MMDH pill**

**+NaIO3**

**CQ+NaIO3**

**CQ+MMDH**

**Pill+NaIO3**
