# Supplementary material for: Ming-Mu-Di-Huang-Pill Activates SQSTM1 via AMPK-Mediated Autophagic KEAP1 Degradation and Protects RPE Cells from Oxidative Damage
Source: Oxid Med Cell Longev. 2022 Mar 25;2022:5851315. doi: 10.1155/2022/5851315 (PMC8976466; doi:10.1155/2022/5851315)
Supplement: Supplementary 5 — Supplemental Table 1: the detailed information of identified components in positive ion mode and negative ion mode by UHPLC-ESI-Q-TOF-MS/MS. [file 5851315.f5.doc]

Supplemental Table1. The detailed information of identified components in positive ion mode and negative ion mode byUHPLC-ESI-Q-TOF-MS/MS.

| No. | Retention time (min) | Precursor ion form | Molecular formula | Measured mass (Da) | Predicted mass (Da) | Mass Error(ppm) | Identification |
| --- | --- | --- | --- | --- | --- | --- | --- |
| 1 | 1.6399 | [M+H]^+^ | C15H10O7 | 303.0497 | 302.24 | 0.002679 | quercetin |
| 2 | 2.634467 | [M+H]^+^ | C15H24O9 | 371.1313 | 348.34 | 0.065428 | Ajugol |
| 3 | 0.881958 | [M+H]^+^ | C17H26O10 | 408.1871 | 390.38 | 0.045615 | Loganin |
| 4 | 0.728588 | [M+H]^+^ | C11H14O5 | 227.0913 | 226.23 | 0.003807 | Sarracenin |
| 5 | 1.4759 | [M+H]^+^ | C15H12O5 | 273.0758 | 272.25 | 0.003033 | Naringenin |
| 6 | 0.696052 | [M+H]^+^ | C6H6O3 | 127.0389 | 126.11 | 0.007366 | 5-Hydroxymethylfurfural |
| 7 | 0.61946 | [M+H]^+^ | C7H6O2 | 123.0442 | 122.12 | 0.007568 | Benzoic acid |
| 8 | 0.553985 | [M+H]^+^ | C5H14NO | 104.1069 | 104.17 | -0.00061 | Choline [M]+ |
| 9 | 7.17925 | [M+H]^+^ | C32H48O6 | 529.3512 | 528.72 | 0.001194 | Alisol C 23-acetate |
| 10 | 4.189117 | [M+H]^+^ | C15H10O6 | 287.0548 | 286.24 | 0.002846 | Kaempferol |
| 11 | 15.47033 | [M+H]^+^ | C5H11NO2 | 118.0862 | 117.15 | 0.007991 | Betaine |
| 12 | 0.728278 | [M+H]^+^ | C16H18O9 | 355.1024 | 354.31 | 0.002237 | Chlorogenic Acid |
| 13 | 2.390083 | [M+H]^+^ | C15H10O6 | 287.0552 | 286.24 | 0.002848 | Luteolin |
| 14 | 5.233592 | [M+H]^+^ | C12H14O2 | 191.1064 | 190.24 | 0.004554 | Ligustilide |
| 15 | 2.409267 | [M+H]^+^ | C9H8O4 | 181.0498 | 180.16 | 0.004939 | Caffeic acid |
| 16 | 2.4443 | [M+H]^+^ | C23H28O11 | 503.1528 | 480.46 | 0.047231 | albiflorin |
| 17 | 0.836165 | [M+H]^+^ | C34H28O22 | 806.1438 | 788.57 | 0.022286 | [(2R,3R,4S,5R,6S)-2-(hydroxymethyl)-4,5,6-tris[(3,4,5-trihydroxybenzoyl)oxy]oxan-3-yl] 3,4,5-trihydroxybenzoate |
| 18 | 9.2011 | [M+H]^+^ | C19H38O4 | 313.2736 | 330.5 | -0.05212 | 2,3-dihydroxypropyl hexadecanoate |
| 19 | 2.819192 | [M-H]^-^ | C23H28O11 | 479.1563 | 480.46 | -0.00271 | Paeoniflorin |
| 20 | 0.72291 | [M-H]^-^ | C17H26O11 | 451.1459 | 406.38 | 0.110158 | Morroniside |
| 21 | 2.35155 | [M-H]^-^ | C15H10O7 | 301.0353 | 302.24 | -0.00399 | Quercetin |
| 22 | 11.68228 | [M-H]^-^ | C23H34O5 | 389.2373 | 390.51 | -0.00326 | DIGOXIGENIN |
| 23 | 15.86996 | [M-H]^-^ | C18H32O16 | 503.1612 | 504.44 | -0.00254 | Manninotriose |
| 24 | 0.879381 | [M-H]^-^ | C12H22O11 | 341.1089 | 342.3 | -0.00348 | Sucrose |
| 25 | 1.546253 | [M-H]^-^ | C21H22O10 | 433.1154 | 434.39 | -0.00293 | Flavanone + 3O, O-Hex |
| 26 | 3.0048 | [M-H]^-^ | C15H12O7 | 303.051 | 304.25 | -0.00394 | Taxifolin |
| 27 | 1.117989 | [M-H]^-^ | C21H20O12 | 463.0893 | 464.38 | -0.00278 | Quercetin-3-O-galactoside |
| 28 | 1.155835 | [M-H]^-^ | C7H6O5 | 169.0143 | 170.12 | -0.0065 | Gallic acid |
| 29 | 3.076658 | [M-H]^-^ | C8H8O5 | 183.0298 | 184.15 | -0.00608 | Methyl gallate |
| 30 | 0.820152 | [M-H]^-^ | C15H14O6.H2O | 289.0721 | 308.28 | -0.06231 | (+)-Catechin hydrate |
| 31 | 4.232092 | [M-H]^-^ | C45H72O17 | 883.4683 | 885.04 | -0.00178 | Gracillin |
| 32 | 11.86763 | [M-H]^-^ | C33H52O5 | 527.3735 | 528.76 | -0.00262 | Pachymic acid |
| 33 | 9.6526 | [M-H]^-^ | C32H52O6 | 577.3757 | 532.75 | 0.083765 | Alisol A 24-acetate |
| 34 | 8.328533 | [M-H]^-^ | C30H50O5 | 535.3643 | 490.71 | 0.090999 | Alisol A |
| 35 | 8.705717 | [M-H]^-^ | C17H34O2 | 315.2541 | 270.45 | 0.165665 | Methyl hexadecanoate |
| 36 | 4.194025 | [M-H]^-^ | C15H10O6 | 285.0412 | 286.24 | -0.00419 | Kaempferol |
| 37 | 4.433325 | [M-H]^-^ | C16H12O7 | 315.0513 | 316.26 | -0.00382 | Isorhamnetin |
| 38 | 0.66994 | [M-H]^-^ | C16H18O9 | 353.0871 | 354.31 | -0.00345 | CHLOROGENIC ACID |
| 39 | 2.635767 | [M-H]^-^ | C21H20O11 | 447.0936 | 448.38 | -0.00287 | Luteolin-4'-O-glucoside |
| 40 | 4.804283 | [M-H]^-^ | C15H10O5 | 269.0456 | 270.24 | -0.00442 | Apigenin |
| 41 | 4.248717 | [M-H]^-^ | C12H14O4 | 221.0819 | 222.24 | -0.00521 | Ethyl ferulate |
| 42 | 0.936329 | [M-H]^-^ | C9H8O4 | 179.035 | 180.16 | -0.00624 | Caffeic Acid |
| 43 | 1.156269 | [M-H]^-^ | C23H28O11 | 479.1564 | 480.46 | -0.00271 | albiflorin |
| 44 | 0.831007 | [M-H]^-^ | C34H28O22 | 787.0997 | 788.57 | -0.00186 | [(2R,3R,4S,5R,6S)-2-(hydroxymethyl)-4,5,6-tris[(3,4,5-trihydroxybenzoyl)oxy]oxan-3-yl] 3,4,5-trihydroxybenzoate |
| 45 | 0.355598 | [M-H]^-^ | C16H32O2 | 255.2333 | 256.42 | -0.00463 | ISOPALMITIC ACID |
